# Single-cell transcriptomics reveals lineage trajectory of human scalp hair follicle and informs mechanisms of hair graying

**DOI:** 10.1038/s41421-022-00394-2

**Published:** 2022-05-24

**Authors:** Sijie Wu, Yao Yu, Caiyue Liu, Xia Zhang, Peiying Zhu, You Peng, Xinyu Yan, Yin Li, Peng Hua, Qingfeng Li, Sijia Wang, Liang Zhang

**Affiliations:** 1 CAS Key Laboratory of Computational Biology, Shanghai Institute of Nutrition and Health, University of Chinese Academy of Sciences, CAS, Shanghai, China; 2 Human Phenome Institute, Fudan University, 825 Zhangheng Road, Shanghai, China; 3 State Key Laboratory of Genetic Engineering and Ministry of Education Key Laboratory of Contemporary Anthropology, Collaborative Innovation Center for Genetics and Development, School of Life Sciences, Fudan University, Shanghai, China; 4 CAS Key Laboratory of Tissue Microenvironment and Tumor, Shanghai Institute of Nutrition and Health, University of Chinese Academy of Sciences, CAS, Shanghai, China; 5 Department of Plastic & Reconstructive Surgery, Shanghai Ninth People’s Hospital, Shanghai Jiao Tong University School of Medicine, 639 Zhizaoju Road, Shanghai, China; 6 Center for Excellence in Animal Evolution and Genetics, Chinese Academy of Sciences, Kunming, China; 7 Institute for Stem Cell and Regeneration, Chinese Academy of Sciences, 1 Beichen West Road, Chaoyang District, Beijing, China

**Keywords:** Skin stem cells, Transcriptomics

## Abstract

Hair conditions, such as hair loss and graying, are prevalent human conditions. But they are often poorly controlled due to our insufficient understanding of human scalp hair follicle (hsHF) in health and disease. Here we describe a comprehensive single-cell RNA-seq (scRNA-seq) analysis on highly purified black and early-stage graying hsHFs. Based on these, a concise single-cell atlas for hsHF and its early graying changes is generated and verified using samples from multiple independent individuals. These data reveal the lineage trajectory of hsHF in unprecedented detail and uncover its multiple unexpected features not found in mouse HFs, including the presence of an innerbulge like compartment in the growing phase, lack of a discrete companion layer, and enrichment of EMT features in HF stem cells (HFSCs). Moreover, we demonstrate that besides melanocyte depletion, early-stage human hair graying is also associated with specific depletion of matrix hair progenitors but not HFSCs. The hair progenitors’ depletion is accompanied by their P53 pathway activation whose pharmaceutical blockade can ameliorate hair graying in mice, enlightening a promising therapeutic avenue for this prevalent hair condition.

## Introduction

Hair conditions, such as hair loss and hair graying, are among the most prevalent human conditions. But they are often poorly controlled since how human hair follicle (HF) functions in disease and health is not well understood. Human hair graying is normally a progressive process starting from middle age. Aging-related hair graying is linked with melanocyte and melanocyte stem cell (MeSC) depletion^1^, which can be affected by factors in the skin microenvironment, such as oxidative stress^2,3^ and neuroendocrine signals^4,5^. Notably, graying hairs also differ from their pigmented peers in growth rate and biophysical properties^5–7^, indicating changes in their HF epithelial lineage. This coincident with findings in mouse showing that epithelial stem cells (SCs) in HF provide a functional niche for MeSCs and instruct their differentiation^8,9^.

Seminal studies have established a comprehensive view for the organization and regeneration of HF epithelial lineage in mouse. In brief, progenies of stem cells in HF bulge and hair germ (HG) contribute to HF regeneration by differentiating into HF outer root sheath (ORS) and transit-amplifying cells (TAC) in the HF matrix. The TACs then differentiate into multiple lineages that form the hair shaft and its surrounding structures^10–13^. However, human HFs distinct from mouse HFs in many ways. For example, the HF growth phase can last for years in human but only weeks in mouse^14^. Human HFs’ growth is largely independent of their neighbors, unlike the coordinative growth waves in mouse^15^. The effect of androgens on HF growth is much more prominent in human than in mouse^15^. Molecularly, putative human HF stem cells (hHFSCs) are found to express different marker genes in comparison with mouse HF stem cells (mHFSCs)^16^.

The advent of single-cell RNA-sequencing (scRNA-seq) technology greatly facilitated the study of complex tissues. scRNAseq of mouse skin not only confirmed classic models of mouse HF homeostasis and regeneration but also provided unprecedented details on these processes^17–19^. Recently, scRNA-seq analysis on whole human scalp micrografts was performed^20^. However, the micrografts used for the scRNA-seq study contained not only HFs but also interfollicular epidermis (IFE), dermis and a variety of other tissues^20^, making it very difficult to delineate human HF’s lineages in these data.

In this study, we describe a comprehensive scRNA-seq analysis on highly purified human scalp HF (hsHF) without contaminations from other tissues. Importantly, we also separated adjacent black and gray hsHFs from the same individual with grizzled hair and analyzed them in parallel. With these data, we generated a concise single-cell atlas for hsHF and its early graying changes, revealed unexpected lineage trajectories and stem cell prosperities in hsHF, and uncovered a therapeutically targetable mechanism underlying hair graying.

## Results

### Single-cell RNA-seq reveals lineage trajectory of hsHF

**Fig. 1:**
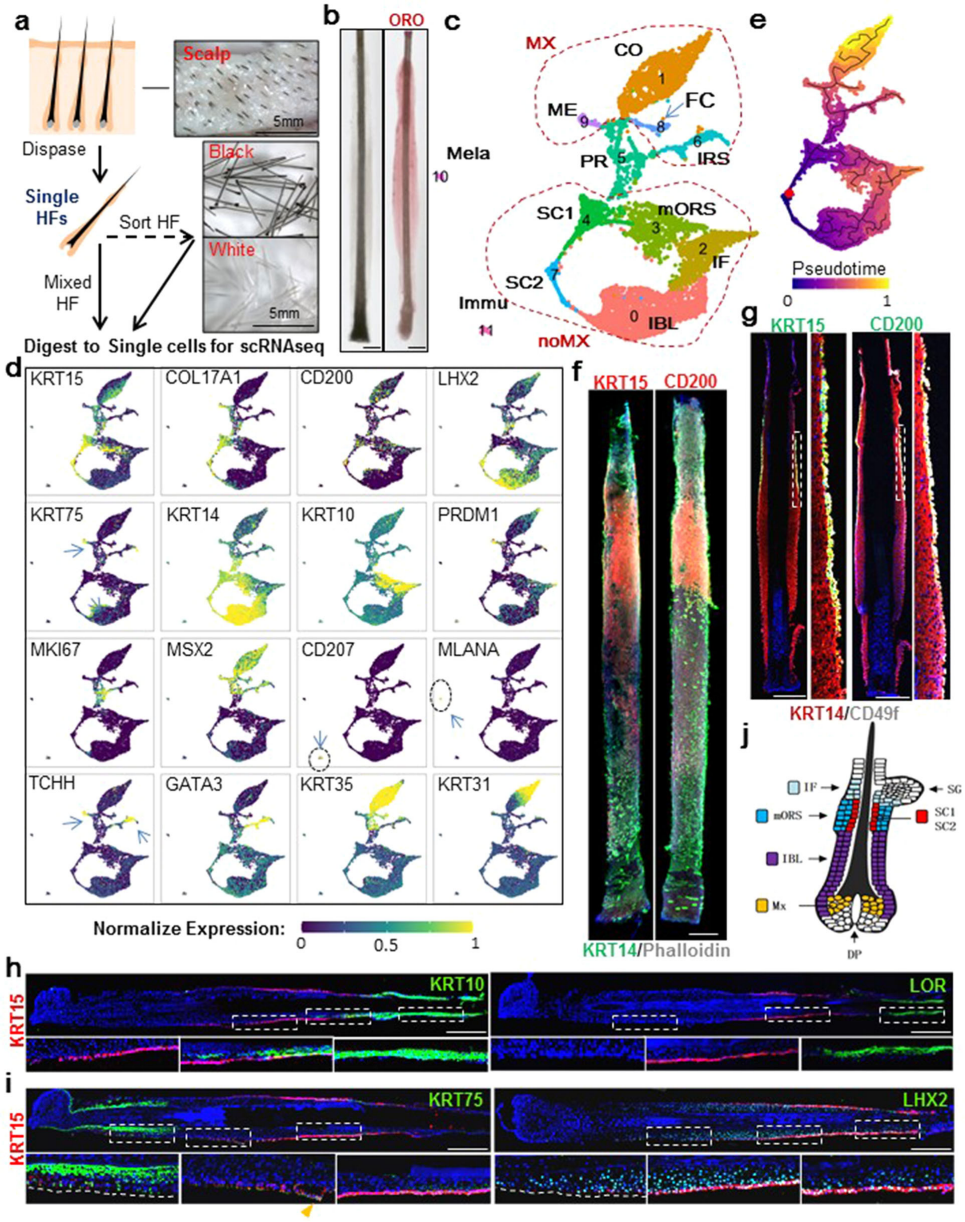
Single-cell RNA-seq reveals cell-type composition of hsHF. **a** Paradigm of isolating hsHF and preparing its single-cell suspension. **b** White field images of single-HFs that are unstained or oil red O (ORO) stained. Scale bars: 200 μm. **c** Uniform manifold approximation and projection (UMAP) plot showing 12 cell types of hsHF. Cell types are 0-11 numbered and colored. Cell-type acronyms are annotated as following: SC1 and SC1: two groups of putative HFSCs (hair follicle stem cells), IBL "inner bulge" like layer, IF infundibulum, PR proliferating progenitors, mORS middle outer root sheath, IRS inner root sheath, ME medulla, CO cortex, FC fibre cuticle of hair shaft, MX matrix compartments, noMX epithelial compartments outside matrix, Mela melanocyte, Immu immune cells. **d** Representative marker genes expression visualized on UMAP. Arrows indicate positive cells in a graph. To avoid occlusion, cells with high expression are drawn preferentially at the top. **e** UMAP visualization of major trajectories and monocle3 pseudo-time of epithelial cell types. The root cell was indicated in red. Cell color is depicted from dark purple to light yellow according to the pseudotime. Edges in the principal graphs that define trajectories reported by Monocle 3 are shown as black line segments. **f** Representative (*n* = 5 HFs from 3 individuals, which are F50, F55, F57. The same 3 individuals’ samples were used in all following hsHF stainings unless otherwise noted) whole mount IF staining image of hsHFs. Scale bars: 200 μm. Blue signal in IF images is DAPI, same in all following figures unless otherwise mentioned. **g**, **h**, **i** Representative IF images of hsHF sections and magnified views of their squared areas. When there are multiple squared areas and magnified views in a single panel, they correspond to each other in a sequential manner, same in all following similar figures. Scale bars: 200 μm. For **g**, *n* = 7 HFs from 3 individuals. For **h**, *n* = 9 HFs from 3 individuals for the left panel and *n* = 7 HFs from 3 individuals for the right panel. For **i**, *n* = 5 HFs from 3 individuals. **j** Sketchy map for the spatial organization of different cell populations on human scalp anagen HF.

We obtained discarded human scalp skin samples from plastic surgery and isolated HFs from them by dispase digestion followed by individually pulling the HFs out of dermis (Fig. 1a). The vast majority of the hsHFs isolated by this method displayed elongated shapes resembling the growth phase (anagen) (Fig. 1a). Closer examination showed that no sebaceous gland (SG), interfollicular epidermis (IFE) or dermis was attached to these isolated hsHFs (Fig. 1b). The isolated hsHFs were further digested into single-cell suspension for scRNA-seq using 10× technology^21^.

Scalp samples were collected from Chinese females at age 18 (F18, predominantly black hair), age 31 (F31, grizzled hair), age 59 (F59, predominantly black hair), and age 62 (F62, grizzled hair), respectively. The hsHFs from F18 and F59 black hair samples were collected and sequenced directly. The hsHFs were further sorted into black hsHFs (F31B or F62B) and white hsHFs (F31W or F62W), and sequenced respectively (Fig. 1a, Supplementary Table S1).

After stringent cell filtration, totally 14,169 cells from the six hsHF samples were retained for subsequent analysis. Following a standard scRNAseq data analysis paradigm^22^, the samples were integrated together via CCA (Seurat)^22^ method, clustered into 12 distinct cell groups (C0-C11), and projected in a two-dimensional space of uniform manifold approximation and projection (UMAP) (Fig. 1c, please also see materials and methods for details of sample QC and integration process). Using modularity, which was defined as the (scaled) difference between the observed total weight of edges between nodes in the same cluster and the expected total weight if edge weights were randomly distributed across all pairs of nodes^23^, all the cells clusters were found to be sufficiently separated from each other (Supplementary Fig. S1a). The 12 cluster groups could be reproduced using cells from each of the 6 samples, suggesting that they are robustly present across different samples (Supplementary Fig. S1b).

The 12 groups’ cellular identities were sketchily characterized based on their expression of known lineage markers (Fig. 1c). C10 and C11 lack keratin expression but are enriched for melanocyte marker *MLANA*^24^ and Langerhans cell marker *CD207*^25^ respectively (Fig. 1d), suggesting that they are melanocytes and immune cells respectively. C0-C9 all display strong keratin expressions, indicating that they are all HF epithelial cells (Fig. 1d). The 10 epithelial cell groups are separated into two major branches with a C5 group in the middle (Fig. 1c). C5 is the only group enriched with cell proliferation marker *MKi67*^26^ (Fig. 1d), suggesting that it represents proliferating progenitors (PR). The upper branch (Mx) includes C9, C1, C8, and C6. They are all highly enriched for HF matrix marker *MSX2*^27^ (Fig. 1d), indicating that they represent the HF matrix lineage. These Mx groups can be further classified based on known hair differentiation markers^19,28–30^ (Fig. 1d). Specifically, C1 is likely hair cortex (CO) based on *KRT31* expression. C6 is likely IRS based on *GATA3* and *TCHH* (AE15) double-positive expression. C9 is likely Medulla (ME) since it expresses only *TCHH* but not *GATA3*. C8 is likely hair fibre cuticle (FC) since it expresses *KRT35* but lacks *KRT31*.

The lower branch (noMx) includes C0, C2, C3, C4, C7 (Fig. 1c). They lack *MSX2* and hair differentiation markers but most of them are enriched for *KRT14(K14)*, which is mainly expressed by ORS cells in HF. Among them, C4 and C7 are likely hHFSCs (named SC1 and SC2 respectively), since they are highly enriched for multiple known hHFSC markers, including *KRT15 (K15)*, *COL17A1*, and *CD200*^8,16,31^, and also lack all the major differentiation markers examined (Fig. 1d). The identities of other noMx groups are relatively obscure. C3 is a *KRT14* enriched group with reminiscent *KRT15* expression and is closely connected to the hHFSCs, suggesting that it likely represent middle ORS cells (mORS) adjacent to hHFSCs. Surprisingly, it also expressed a significant amount of *KRT10*, which is an IFE differentiation marker that is normally absent from mouse HFs^32^. C0 expresses not only *KRT14* but also *KRT6C*, *KRT75 (K6hf)*, and *LHX2* (Fig. 1d, Supplementary Fig. S1e). We named it IBL since we speculate that it is similar to the “innerbulge” cells in mice^33^. C2 is enriched for IFE differentiation marker *KRT10(K10)* and also SG precursor marker *PRDM1(BLIMP1)*^34^ (Fig. 1d). It likely represents regions near the infundibulum (IF), which is adjacent to IFE and SG.

To investigate the differential trajectories among the 10 HF epithelial groups, pseudotime analysis using monocle3^35^ was employed with the root point setting at the junction of two HFSC groups (Fig. 1e). Our data show that the SC1 first differentiates into mORS cells and TA cells, which then further branches into multiple matrix lineages that contribute to hair formation. This pattern fits well with knowledge from seminal mouse studies^10,19^. Interestingly, SC2 appears to have a differentiation route that directly leads to the IBL cells, implicating the complexity of hHFSCs. By GO enrichment analysis^36,37^, SC1, SC2, and PR were found to be the only HF epithelial cell groups that are not enriched for genes related to epithelial cell differentiation (e.g. Cornification, Keratinocyte Differentiation, Epidermis Development) (Supplementary Fig. S1c, Table S2), further supporting that SC1 and SC2 are the only undifferentiated hHFSC populations. In comparison with SC2, SC1 appeared to be relatively enriched for genes involved in gene expression machinery while having comparable data quality (Supplementary Fig. S1c, d), suggesting that SC1 is likely a relatively more active hHFSC population that is poised for proliferation. On the other hand, SC2 is likely a more quiescent group since it is enriched for *FGF18* (Supplementary Fig. S1e), a key signal for maintaining HFSC quiescence in mouse^33^.

To determine the spatial location of these hsHF cell groups, we conducted a serial of IF stainings on isolated hsHFs. Whole-mount stainings showed that hHFSC marker KRT15 and CD200 were enriched in hsHF upper-middle part (Fig. 1f), an expected “bulge” position. IF staining of hsHF sections showed that KRT15 and CD200 protein expression were highly restricted to the outermost layer of upper-middle ORS, while KRT14 was present ubiquitously in all ORS layers (Fig. 1g), suggesting that hHFSCs in growing hsHFs are restricted to the outermost layer of upper-middle ORS. In addition, the KRT15 staining positive cells were mostly negative for Ki67 staining, consistent with the expected quiescent nature of HFSCs (Supplementary Fig. S1f). 2-D plot of *KRT15* vs *Ki67* mRNA expression also showed that cells with high-level *KRT15* transcripts were mostly low or negative for *Ki67* transcripts (Supplementary Fig. S1h).

Consistent with the scRNA-seq data, significant KRT10^+^ expression was detected by IF in the upper ORS area above the KRT15^+^ region and extended to the suprabasal layers of the upper part of the KRT15^+^ region (Fig. 1h), with a small amount of KRT10^+^KRT15^+^ cells present in the transition area (Supplementary Fig. S1h). The KRT10^+^ expression in this upper ORS area was comparable to the KRT10 signal in the epidermis (Supplementary Fig. S1h, i). In contrast, late IFE differentiation marker Loricrin (*LOR*) was absent from the KRT15^+^ cells' nearby regions (Fig. 1h). These data suggest that the mORS population containing both *KRT14*^*+*^ and *KRT10*^*+*^ cells is likely middle to upper region ORS cells. Similarly, significant KRT75 expression was detected by IF in the lower ORS area below the KRT15^+^ region (Fig. 1i). Interestingly, the KRT75 expression spreads into multiple layers of the lower ORS region rather than confines to a single companion layer as it does in mouse anagen HF (Supplementary Fig. S1j). Some of the KRT75 staining even extended to KRT15^+^ cells in the lower HFSC region (Fig. 1i). In addition, cells in the middle to lower ORS areas were mostly positive for LHX2 staining as well (Fig. 1i). These data suggest that the *KRT75*^*+*^*LHX2*^*+*^ IBL population likely represents cells in middle to lower ORS region.

Taken together, we concluded a sketchy map for the spatial organization of different cell populations of anagen hsHF (Fig. 1j). It has both similarity and significant dissimilarity in comparison with that of mouse HFs.

### Human scalp HFSCs display different molecular features from their Murine counterparts

**Fig. 2:**
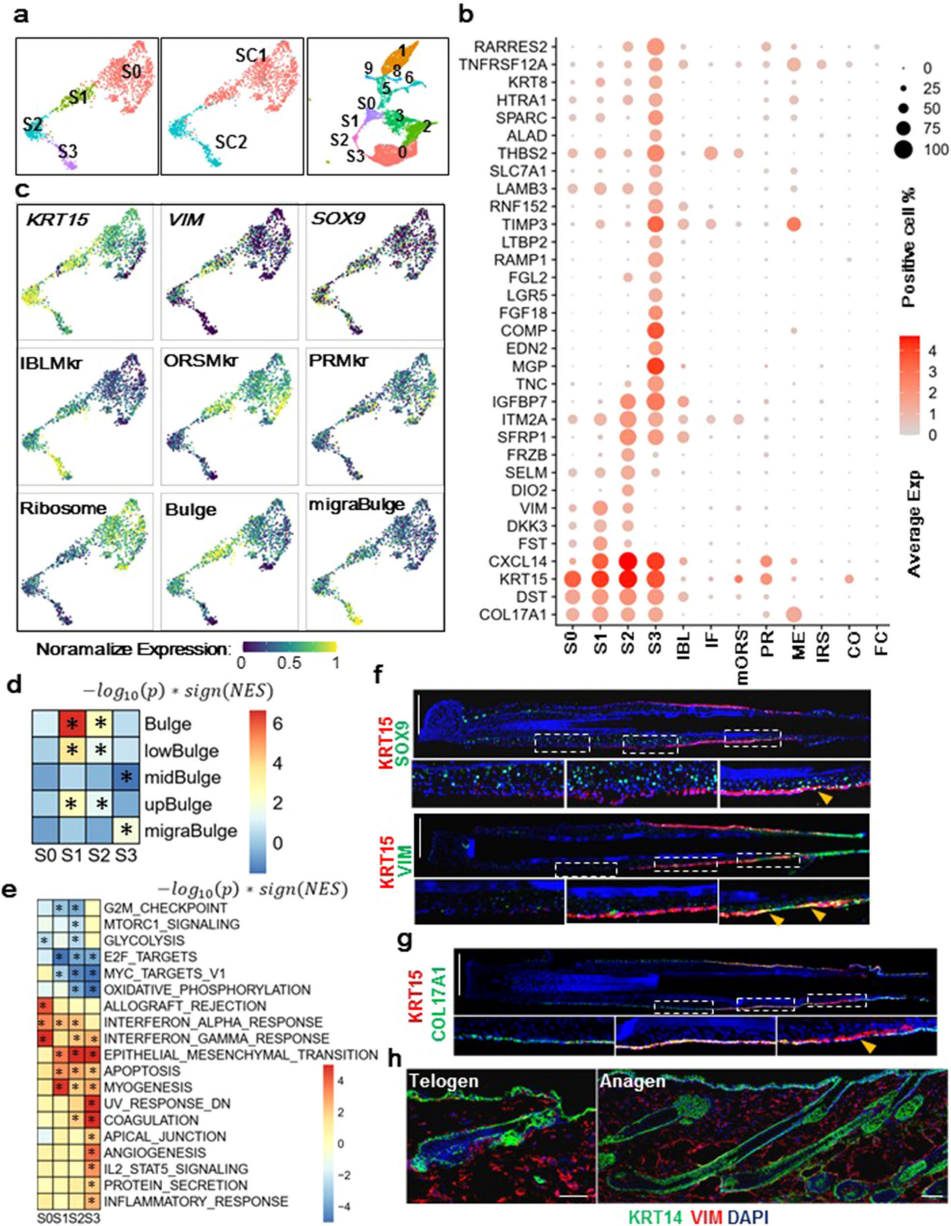
Human scalp HFSCs display different molecular features from their Murine counterparts. **a** UMAP plots showing the subpopulations of Hair follicle stem cells (SC1 + SC2). **b** Dot plot showing the expression of human HFSC signature genes. **c** Representative marker genes expression visualized on UMAP. IBLMkr, ORSMkr, and PRMkr: marker gene of IBL, mORS, and PR group respectively. Bulge & migraBulge: previously reported mHFSC subgroups signature genes for core bulge and migrating bulge respectively. See also Supplementary Tables S4. **d** Heatmap showing GSEA analysis results of previously characterized mHFSC subgroups markers (vertical axis) in the HFSCs subpopulations (horizon axis). Red indicates enrichment while blue indicate depletion. NES: normalized enrichment score. *P < 0.05. **e** Heatmap showing the GSEA analysis results of MSigDB hallmark terms in the HFSC subpopulations. *P < 0.01. **f** Representative (*n* = 5 HFs for the upper panel and *n* = 8 HFs from 3 individuals for the lower panel) IF images of hsHF sections and magnified views of squared areas. Scale bars: 200 μm. **g** Representative IF images (*n* = 5 HFs from 3 individuals) of hsHF sections and magnified views. Scale bars: 200 μm. **h** Representative (*n* = 3) IF images WT mouse telogen (P50)/anagen (2 weeks post depilation at P50) HF sections. Scale bars: 50 μm.

We further interrogated potential heterogeneity among human HFSCs (hHFSCs) by subclustering the SC1 plus SC2 populations. Four subclusters (S0, S1, S2, S3) were identified and each of them roughly corresponded to a portion of the SC1 (S0 + S1) and SC2 (S2 + S3) groups (Fig. 2a). We conducted a Wilcoxon rank-sum test to characterize genes that were specifically enriched in anyone of these four subpopulations versus hHFSC neighbors on UMAP including mORS, IBL, and PR. Totally 33 HFSC enriched genes (hHFSC-Sig) were identified based on a criteria of avg_AUC (average of AUC) > 0.8, pct_dff (difference in the fraction of detection between the two groups) > 0.3 and FDR < 0.05 (Fig. 2b, Supplementary Table S3). All of them were robustly reproduced in each of the 6 individual samples (Supplementary Fig. S2a).

The hHFSC-Sig included *KRT15* and *COL17A1*, two known hHFSC markers. Another known hHFSC marker *CD200* was visibly enriched in S2 but failed to meet the hHFSC-Sig criteria likely due to its low expression level (Supplementary Fig. S2b). By IF stainings, significant overlap between COL17A1 and KRT15 in hHFSC region was confirmed (Fig. 2g). Notably, KRT15^+^ but COL17A1^−^ cells were visible in middle to upper ORS region (Fig. 2g), which may represent the *KRT15*^+^ cells in the mORS group (Fig. 1c).

Interestingly, many key mHFSC markers were missed from the hHFSC-Sig, including *SOX9*, *TCF3*, *TBX1*, *RUNX1*, *NFATC1*, *LHX2*, and *CD34*^38–40^. We examined their expression patterns individually (Supplementary Fig. S2b). Among these, *NFATC1* was visibly enriched in S1 and S2, while *LHX2*, *CD34* and *TBX1* appeared to be enriched in S3. They were likely genes enriched in a subset of hHFSCs but failed to meet the hHFSC-Sig criteria due to low expression. On the other hand, *SOX9* is a key mHFSC marker and master regulator that is abundantly expressed in all mHFSCs and some of their progenies^32,41,42^. But in the hsHFs, its transcript was only weakly expressed in a small portion of hHFSCs (Fig. 2c, Supplementary Fig S2b). Consistently, IF stainings showed that SOX9 protein are mostly enriched in the lower ORS region, while only a small potion KRT15^+^ cells have detectable SOX9 expression (Fig. 2f).

A surprising gene in the hHFSC-Sig is *VIM*, which encodes Vimentin that is normally expressed in mesenchymal cells but not in skin epithelial cells^43–45^. Our IF staining of mouse skin sections also confirmed the absence of *VIM* from all skin epithelial cells in both growing and resting stages (Fig. 2h). Interestingly, IF staining of hsHF sections detected clear VIM signal in a subset of KRT15^+^ cells in the upper HFSC region (Fig. 2f). This was consistent with the scRNAseq data showing that *VIM* expression was mostly enriched in S1 and S2 cells (Fig. 2b).

The S0-S3 subgroups likely represent heterogeneous hHFSC populations in vivo. S3 and S0 groups were enriched for IBL markers and mORS markers respectively (Fig. 2c), suggesting that they have different differentiation tendency. When compared with previously characterized mHFSC subgroups via scRNA-seq^18^, the S3 group was specifically enriched for migrating bulge (migroBulge) signature genes (Fig. 2d, Supplementary Table S4), implicating that it represented HFSCs migrating out of their niche. On the other hand, mouse core bulge signature genes^18^ were mostly enriched in S1 and S2, suggesting that they likely represented quiescent hHFSCs (qHFSCs). Interestingly, S1 and S2 were also the groups enriched for *VIM*, which was a classic EMT marker^46,47^ (Fig. 2c). Additional GSEA analysis using Msigdb hallmark genes^48,49^ also revealed significant enrichment of EMT hallmarks in S1-S3 (Fig. 2e). These data implicated an interesting possibility that EMT might be involved in hHFSC fate regulation. In addition, the S0 group was enriched for ribosome genes (Fig. 2c) and was closely linked to proliferating cells on UMAP (Fig. 2a), suggesting that it represented hHFSCs poised for activation. Interestingly, the S0 subgroup was also highly enriched for hallmarks of the immune response, which might be reminiscent to the importance of immune cells in HFSC activation^50^.

We also compared the hsHF data with a previously published human eyelid skin (EIS) scRNA-seq dataset^51^. The hsHF and eyelid skin data were integrated and clustered together on a UMAP (Supplementary Fig. S2c). The SC1 subgroup of hHFSC was closely clustered to the eyelid IFE basal cells but the SC2 subgroup was distinct from any IFE cells (Supplementary Fig. S2c). Notably, ribosome gene expression in SC1 but not SC2 is comparable to that of IFE basal cells (Supplementary Fig. S2d). These are consistent with the idea that SC1 may represent a relatively active stem cell population similar to IFE stem cells. The mORS group was closely clustered to eyelid IFE supra-basal cells with comparable level of *KRT10* expression (Supplementary Fig. S2c, d, S1h). Expression of *VIM* was detected in hHFSCs but not in eyelid IFE basal cells, at a level lower than that of mesenchymal cells (Supplementary Fig. S2c, d). This is confirmed by IF stainings on section of human scalp micrografts with dermal tissues (Supplementary Fig. S2e). These data reinforced the idea that *VIM* expression is a feature of hHFSCs.

### Human matrix TAC contains heterogeneous populations with distinct differentiation tendency

**Fig. 3:**
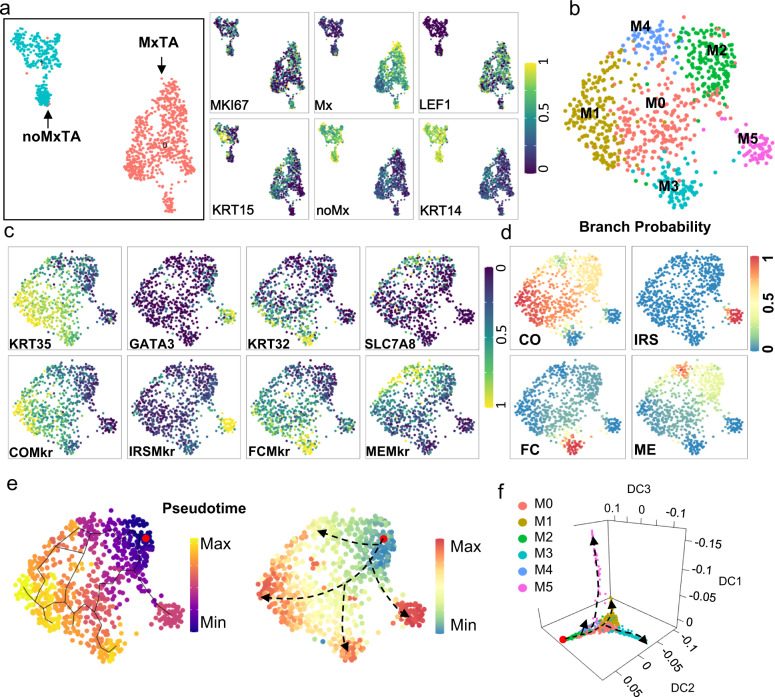
Human HF TAC contains heterogeneous populations with distinct lineage tendencies. **a** Subclustering of PR cells and marker genes expression visualized on UMAP. **b** Subclustering UMAP of MxTA cells from **a**. **c** Representative marker genes expression visualized on MxTA subclustering UMAP. COMkr: CO markers; IRSMkr: IRS markers; FCMkr: FC markers; MEMkr: ME markers. **d** Palantir branch probabilities for CO, IRS, FC, and ME cell lineages in Matrix TA cells. Cells were colored according to their Palantir branch probabilities. **e** UMAP visualization of Matrix TA cells, but with cells now colored according to their monocle3 pseudo-time (left) and diffusion pseudo-time (right), respectively. The root cell was indicated in red. **f** Diffusion map of the single cells along the top three diffusion components (DC). Cells were colored according to subclusters in **b**. The root cell was indicated in red and putative lineage trajectories displaying the bifurcation into CO, IRS, FC, and ME lineages were shown in black.

Subclustering of the proliferating (PR) cells revealed two highly distinct subgroups (Fig. 3a). One was enriched for matrix (Mx) signature genes and *LEF1*, representing transient amplifying cells (TACs) in the matrix (Fig. 3a). We named it MxTA. The other one lacked matrix markers but was enriched for ORS marker *KRT14* and also contained a substantial amount of *KRT15*^+^ cells (Fig. 3a), suggesting that it represented proliferating hHFSCs or their immediate progenies on ORS. We named it noMxTA.

Studies in mouse have shown that matrix TACs contain heterogeneous groups with predisposed differentiation tendencies^19^. To examine this in hsHF, we attempted to further sub-cluster the MxTA cells and detected 6 major subgroups (M0-M5) (Fig. 3b), which displayed highly heterogeneous distribution of different hair lineage markers. Specifically, M1 was enriched for CO markers, M5 was enriched for IRS markers, M3 was enriched for FC markers, and M4 was enriched for ME markers (Fig. 3c). M2 lacked hair differentiation markers but were relatively enriched for hHFSC markers (Supplementary Fig. S3a), suggesting that it represented relatively undifferentiated progenitors.

We further applied Palantir algorithm^52^ to measure cell fate probabilities among the MxTA subgroups. The idea of Palantir is to model trajectories of differentiating cells by treating cell fate as a probabilistic process and leverages entropy to measure cell plasticity along the trajectory. The cell with the lowest signature score of MxMkr (Mx branch marker) in M2 was chosen as the initial cell, and four cells randomly picked from CO, IRS, ME and FC were used as terminal state cells. Consistent with the above findings, this analysis revealed clear differentiation tendency to CO, IRS, ME, and FC for M1, M5, M3, and M4 respectively (Fig. 3d). We also examined the lineage trajectories among MxTA subgroups using monocle3 and Diffusion Maps^53^, which used a distance metric (diffusion distance) to examine how differentiating cells follow moving from a multipotent state towards more differentiated states. The results confirmed that these MxTA subgroups followed a characteristic topology of different lineages branching from a central population of progenitor cells (M2) (Fig. 3e, f). IF stainings also showed an interlaced pattern of TAC markers and differentiation markers in hsHF matrix (Supplementary Fig. S3b), similar to its murine counterpart^19^. Taken together, our findings suggest that human HF matrix TACs is likely organized in a way similar to mouse matrix TACs^19^ in that it likely contains heterogeneous populations with predisposed lineage tendency.

### SCENIC analysis uncovers transcriptional regulation networks in hsHF

**Fig. 4:**
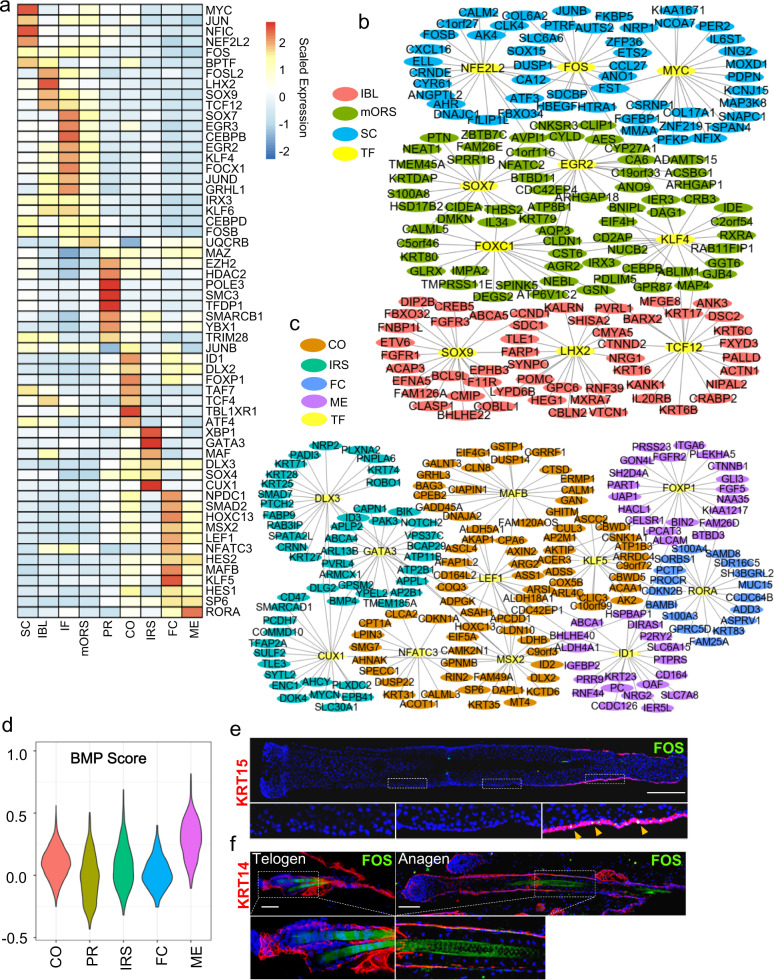
SCENIC analysis uncovers transcriptional regulation networks in hsHF. **a** Heatmap of scaled expression for inferred transcription-factor gene regulatory networks (SCENIC) in 9 cell types of hsHF. **b**, **c** Gene regulatory network analysis using SCENIC identifies critical transcriptional regulation for **(b)** noMx cells or **(c)** Mx cells. The predicted TFs and their target genes are shown. The TFs are highlighted in yellow. **d** Violin plots of canonical *BMP* target gene pathway scores in different cell types. **e** Representative (*n* = 5 HFs from 3 individuals) IF images of hsHF sections and magnified views. Scale bars: 200 μm. **f** Representative (*n* = 3) IF images and magnified views of squared areas on mouse telogen (P50) or anagen (2 weeks post depilation at P50) HF sections (Scale bars: 50 μm).

To gain insights into the transcriptional regulation networks in hsHF, we employed the SCENIC pipeline^54^ to identify significantly enriched gene regulatory networks (regulons) corresponding to specific TFs in each cell group. Since most of the individual hHFSC subgroups had relatively low cell number, we combined S0-S3 into a single hHFSC group (SC) for this analysis. We applied a further filter to remove regulons with low correlation efficacy with corresponding TFs (<0.25) or low TF expression (<0.6). In the end, 56 significant transcriptional regulons were identified (Fig. 4a). Among these, regulons of *MSX2* and *LEF1*, two known key TFs of HF matrix, were enriched in matrix groups as expected. Interestingly, regulons for *SOX9*, *TCF4*, and *LHX2*, three key TFs for mHFSCs, were mostly enriched in IBL cells but not hHFSCs instead. On the other hand, regulons of *JUN* and *FOS*, two AP-1 family TFs^55^ that were not known to participate adult HFSC regulation in mice, were enriched in hHFSCs. These data suggest that transcriptional regulation of hHFSCs may differ significantly from their murine counterparts.

Furthermore, we conducted a Wilcoxon rank-sum test to characterize situations that a TF and its regulon are both specifically and significantly enriched in the same cell groups (avg_logFC > 0.05, regulon.FDR < 0.01, TF.p value < 0.05 and TF.pct.diff > 0.05), and organized them into transcriptional networks (Supplementary Table S5). In the noMx branch, *SOX9* and *LHX2* were found to be the central TFs for IBL group (Fig. 4b). This was consistent with the enrichment of *SOX9* and *LHX2* expression in the IBL cells (Figs. 1i, 2e). On that hand, *MYC*, *NEF2L2*, and *FOS* were found to be the central TFs for hHFSCs (Fig. 4b). By IF stainings, we confirmed that FOS was specifically enriched in a subset of KRT15^+^ hHFSCs (Fig. 4e). Interestingly, IF stainings detected no FOS expression in any part of mouse skin HFs in both telogen and anagen stages (Fig. 4f), suggesting that FOS expression is an hHFSC specific property not shared with mHFSC.

In the matrix branch, *GATA3*, a previously reported key TF for mouse IRS, was also found to be a central TF for human IRS (Fig. 4c). On the other hand, a central TF found for ME lineage was *ID1* (Fig. 4c), which is a critical downstream effector of the *BMP* pathway. Consistently, the ME lineage displayed strongly elevated *BMP* score^56^ in comparisons with other matrix cells (Fig. 4d), implicating the importance of *BMP* signaling in ME differentiation.

### Depletion of Matrix TAC is a major HF change associates with hair graying

**Fig. 5:**
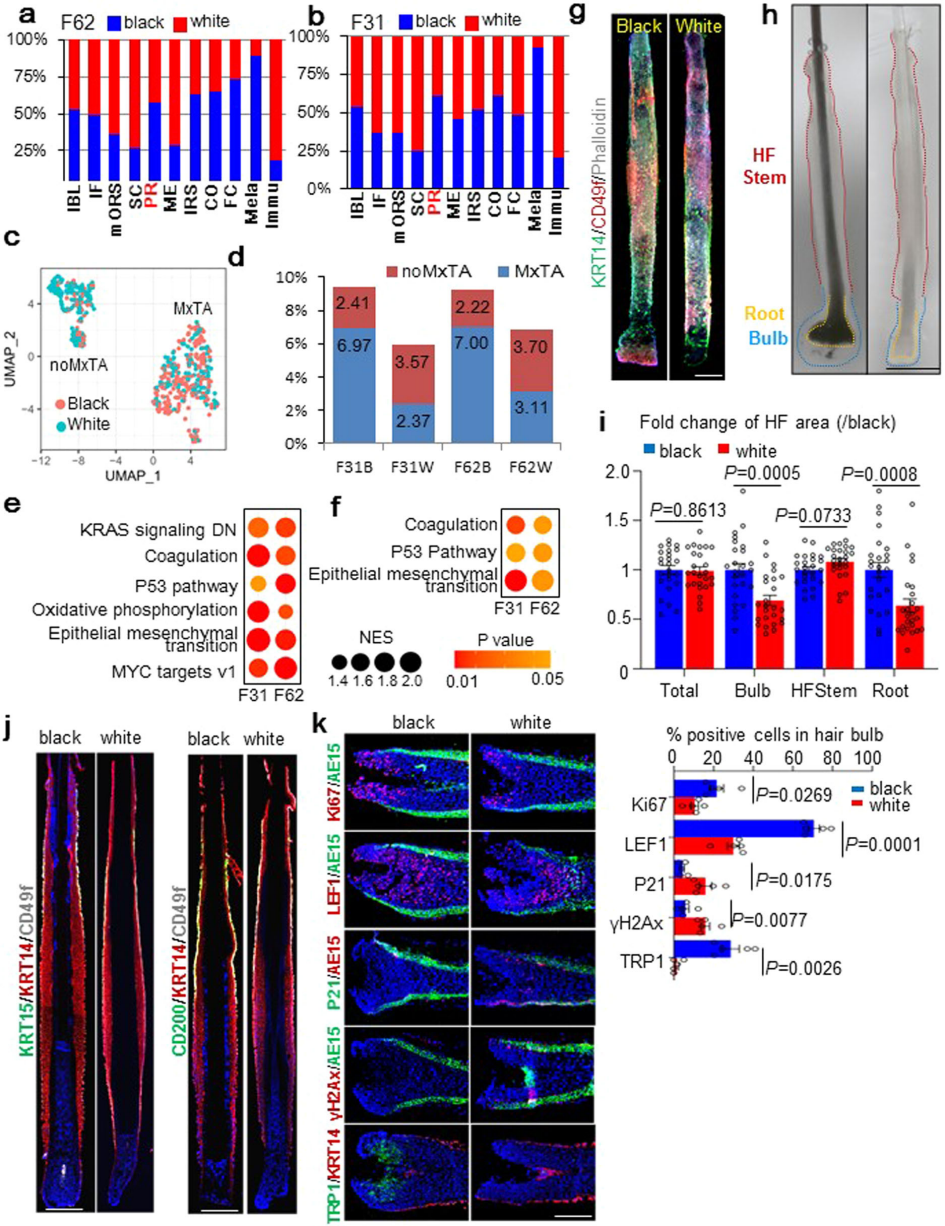
Depletion of Matrix TAC is a major HF change associates with hair graying. **a**, **b** Bar plots of relative abundance of each cell group between white hsHFs and their black neighbors in (**a**) F62 and (**b**) F31 samples. The comparisons were normalized to the total cell numbers of each sample. For example, the F62 IBL bar represents (IBL % in F62B)/(IBL % in F62W). The same applies to all similar figures below. **c** UMAP plots showing the subgroups of proliferating (PR) cells in black (F62B + F31B) and white (F62W + F31W) HFs. **d** Bar plots of noMxTA and MxTA cell % in all HF epithelial cells in each sample. Numbers indicate exact %. **e**, **f** Summary of GSEA analysis result for enriched MSigDB hallmark terms in the F62W-vs-F62B, F31W-vs-F31B gene expression changes in **(e)** MxTAs or **(f)** qHFSCs (S1 + S2). F62W up-regulated terms are displayed. **g, h** Representative (*n* = 5 HFs from 3 individuals) whole mount IF (**g**) or white-field (**h**) images of black and grey hsHFs from the same individuals. Scale bars: 200 μm for **g**, 500 μm for h. Dashed lines label different parts of the HFs. **i** Size quantification of whole HF and its different parts as labeled on **(h)** figure images. (*n* = 25 HF from 3 individuals). **j** Representative (*n* = 5 pairs of black and white HFs from 3 individuals) IF images of human black and white HF sections from the same individuals. Scale bars: 200 μm. **k** Left: Representative (*n* = 5 pairs of black and white HFs from 3 individuals) IF images of human black and white HF matrix sections from the same individuals. Scale bars: 100 μm. Right: Quantification of positive cell % for each of the indicated staining markers in the left panel.

Human hair graying can be attributed to melanocyte depletion, but it was unclear whether there are also HF epithelial lineage alterations involved in this process. To address this question, we conducted pair-wised comparisons in hsHF lineage composition for F62B vs F62W and F31B vs F31W respectively. We reasoned that the comparisons between adjacent black and white hsHFs from the same individuals should specifically reflect lineage changes related to hair graying. As expected, the white hsHFs contained much less melanocytes than the black neighbors in both comparisons (Fig. 5a, b). Interestingly, the white hsHFs also had clearly more immune cells than their black neighbors, implicating elevated immune cell infiltration into hsHFs during hair graying (Fig. 5a, b). In epithelial lineage, it is surprising that the white hsHFs displayed consistently more hHFSCs than their black neighbors in both comparisons (Fig. 5a, b), suggesting that loss of hHFSCs is unlikely the primary cause of human hair graying. When all six hsHF samples were compared in parallel (Supplementary Fig. S4a), it showed that the lineage compositions of hsHF samples from different individuals varied significantly from each other and displayed no obvious pattern, likely due to individual differences and differences in the precise hair cycle status of each scalp sample. This underscores the importance of directly comparing black and white hsHFs from the same scalp sample.

In both F62B-vs-F62W and F31B-vs-F31W comparisons, the white hsHFs displayed slightly less proliferation cells (PR) than their black neighbors (Fig. 5a, b). Interestingly, further analysis of the PR cells showed that the MxTA subgroup was greatly reduced in both of the comparisons, while the noMxTA subgroup was increased instead (Fig. 5c, d), suggesting that matrix TACs were depleted from the white HFs while hHFSC self-renewal was not impaired. These features were not observed for F31B-vs-F62B (Fig. 5d) and F18-vs-F59 comparisons (Supplementary Fig. S4b), suggesting that they are not general features of overall HF aging. Additional analysis of MxTA and hHFSC subgroup compositions detected only one consistent change between F62B-vs-F62W and F31B-vs-F31W comparisons: reduced S3 group (migrating hHFSCs) in white hsHFs (Supplementary Fig. S4c), implicating reduced hHFSC contribution to matrix TAC in white HFs. Next, we conducted a Wilcoxon rank-sum test to look for differentially expressed genes between the white and black hsHFs in each individual cell groups (Supplementary Table S6). By GSEA analysis of the results, significantly elevated hallmarks of P53 pathway activation were detected in both matrix TACs and qHFSCs (S1 + S2) of the white hsHFs vs their black neighbors in both F62B-vs-F62W and F31B-vs-F31W comparisons (Fig. 5e, f), suggesting that P53 pathway activation may contribute to hair graying.

We also conducted systematic analysis of gene expression changes in each cell population in different sample comparison pairs. GSEA analysis showed that F62W-vs-F62B gene expression changes was highly consistent with that of F31W-vs-F31B in most cell populations (Supplementary Fig. S4d), suggesting that hair graying is governed by a common transcriptional mechanism in both relatively young (F31) and relatively old (F62) individuals. In contrast, the HF aging comparisons (F59-vs-F18 and F62B-vs-F31B) displayed little similarity with the hair graying comparisons (F62W-vs-F62B and F31W-vs-F31B) in most cell populations (Supplementary Fig. S4d, Table S7), suggesting that hair graying and overall HF aging are not regulated by identical mechanisms. Additional GSEA analysis using Msigdb hallmark genes confirmed that transcriptional changes in hair graying comparisons (F62W-vs-F62B and F31W-vs-F31B) were similar with each other, but were distinct from that of HF aging comparisons (F59-vs-F18 and F62B-vs-F31B) (Supplementary Fig. S4e).

We further analyzed the melanocyte lineage by re-clustering the hsHF Mela group plus epidermal melanocytes from the published EIS data. Interestingly, these cells clustered evenly without a distinct melanocyte stem cell population (Supplementary Fig. S4f). By GSEA analysis of relative enrichment of MsigDB hallmarks in the melanocytes of each sample, we could not see a clear gene expression pattern for old or young hsHF melanocytes (Supplementary Fig. S4g).

As mentioned above, the hsHF immune cell population was greatly increased in the white hsHFs vs their black neighbors (Fig. 5a, b). The hsHF immune cells could be further clustered into three major subgroups named I0-I2 (Supplementary Fig. S5a). Among these, the relative abundance of the I1 group was increased in both F62W-vs-F62B and F31W-vs-F31B comparisons (Supplementary Fig. S5b). By cell identity annotation using SingleR^57^ and GO enrichment analysis (Supplementary Fig. S5c-e, Supplementary Table S8), the I0 and I2 group were identified as monocytes (likely dendrocytes and macrophages), while the I1 group was identified as CD4^+^ T cells (likely regulatory T cells and T helper cells). These observations implicated an intriguing role of immune cells in hair graying.

Whole-mount imaging of independent samples from other persons confirmed that adjacent white and black hsHFs from the same individuals had comparable length and HFSC marker expressions (Fig. 5g). Only the hair matrix region was significantly miniaturized in the white hsHFs vs adjacent black hsHFs, while other HF parts remained unchanged (Fig. 5h, i). IF stainings on sections of adjacent black and white hsHF samples from other individuals also showed comparable ORS structure and hHFSC marker expression (Fig. 5j), indicating that they are not experiencing HF miniaturization or HFSC loss. In contrast, matrix TAC marker Ki67 and LFF1 alone with melanocyte marker TRP1^58^ were significantly reduced in the matrix of the white hsHFs vs their black neighbors (Fig. 5k). Importantly, P53 pathway target P21^59^ and DNA damage marker histone γH2Ax^60^ were also significantly elevated in the matrix of the white hsHFs vs their black neighbors (Fig. 5k), supporting the idea that P53 pathway is activated in the matrix of graying hsHFs.

### Pharmaceutical inhibition of P53 pathway ameliorate ionizing irradiation-induced hair graying in mice

Previous studies by our group and others showed that HF aging is driven by genotoxic stress-induced HFSC deregulation and can be accelerated by ionizing irradiation (IR) treatment of the skin^61,62^. We further showed that localized IR (LIR) treatment on mouse backskin could be used to model many aspects of skin aging including hair graying^62^. We also showed that LIR aging and physiological skin aging phenotypes are driven by a common molecular mechanism, which is conserved between mouse and human^62^.

**Fig. 6:**
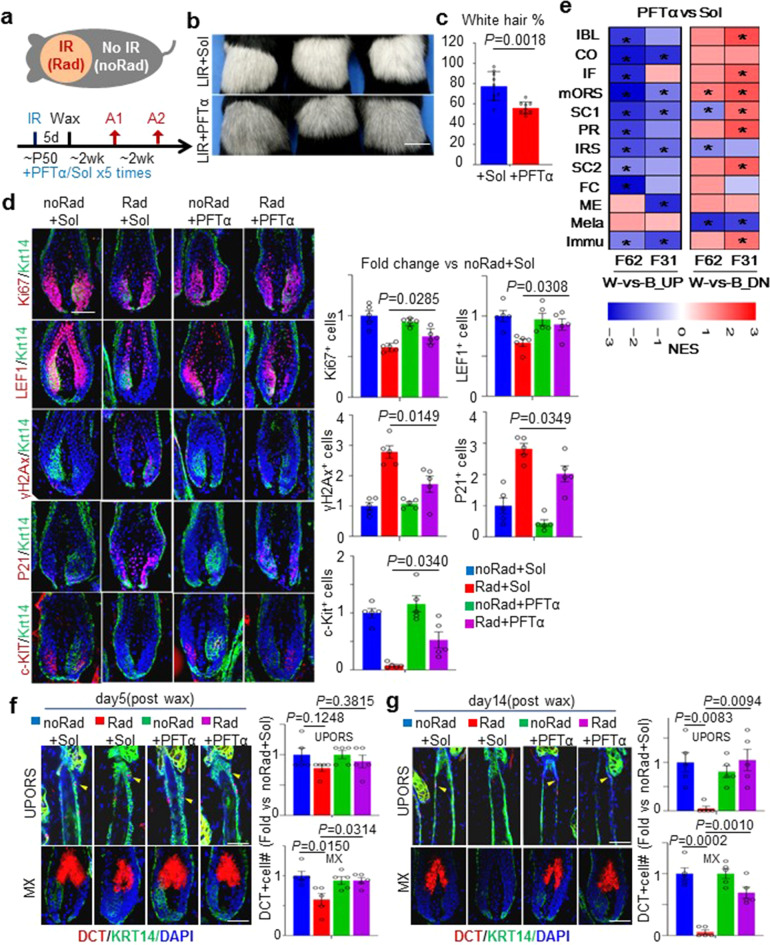
Pharmaceutical inhibition of P53 pathway ameliorate ionizing irradiation-induced hair graying in mice. **a** Schematic of LIR treatment and drug administration. Orange: IR area. PFTα was topically applied daily right after IR for 5 times. Skin samples were collected at 2 weeks post depilation (A1) and photos were taken at 4 weeks post depilation (A2). **b** Backskin images of Rad skin areas of Sol or PFTα treated LIR mice A2 time. **c** Quantification of white hair % of the **b** figure skin area. *n* = 9 sites from 3 mice. **d** Left: Representative (*n* = 3) IF images of mouse HFs matrix region at A1 time. Scale bars: 50 μm. Right: Quantification of positive cell% for each of the indicated staining markers in the HF matrix. Numbers were fold change vs noRad+Sol. all P values were from two-tail *t*-test and all error bars represent standard errors. **e** Heatmap summarizing pre-ranked GSEA analysis results. Input gene sets: significantly up-regulated (UP) or down-regulated (DN) genes in each cell group for white-vs-black (W-vs-B) comparisons of F62 (F62W-vs-F62B) and F31 (F31W-vs-F31B). Ranked lists of genes (Rnk) were produced using gene expression fold change of (Rad+ PFTα skin epithelium) vs (Rad+Sol skin epithelium) as described in the d figure. Red indicated enrichment while blue indicated depletion. NES: normalized enrichment score. *P < 0.05. **f**, **g** Left: Representative (*n* = 3) IF images of LIR mice’s HF upper ORS region around bulge (UPORS) and matrix region(MX). Samples were collected at day 5 (**f**) and day14 (**g**) post the LIR waxing. Right: Quantification of DCT positive cell (arrows) number in UPORS and MX regions as indicated. Scale bars: 50 μm.

Here we employed this model to examine the functional relevance of P53 pathway activation to hair graying. P53 inhibitor drug pifithrin-alpha (PFTα)^63^ or solvent control (Sol) was topically administrated to the LIR mice at 2 μg/cm^2^ dosage every 3 days for 5 times, starting right after the IR treatment and followed their hair graying process thereafter (Fig. 6a). Strikingly, the PFTα treatment significantly reduced hair graying phenotype in the irradiated skin area (Rad) (Fig. 6b, c) without causing discernible changes in untreated skin areas (noRad) (Supplementary Fig. S6a) of the same mice. By IF staining of anagen HFs sections from these mice, we found that Rad skin HFs had significantly reduced matrix TAC marker Ki67 and LFF1, significantly reduced melanocyte marker c-KIT, and significantly increased P53 target P21 and DNA damage marker γH2Ax in their matrix in comparison with noRad skin HFs from the same mice (Fig. 6d). These closely resembled the molecular features of physiologically graying hsHFs described above (Fig. 5k). Importantly, in comparison with Sol control, PFTα treatment significantly suppressed these changes in Rad skin HF matrix, including reducing P21 & γH2Ax expression and increasing Ki67 & LFF1 & c-KIT expression (Fig. 6d). We also systematically analyzed the gene expression changes induced by PFTα treatment in the Rad skin epithelium by RNAseq (Supplementary Table S9). By cross-species GSEA analysis, we showed that the PFTα treatment of mouse Rad skin induced gene expression changes that were mostly opposite to the human hair graying associated changes (Fig. 6e).

We also examined the dynamics of melanocytes and their stem cells (MeSCs) during the first anagen phase of the above PFTα or solvent-treated LIR skin HFs (Fig. 6a). In early anagen(day 5 postwaxing), DCT(Dopachrome Tautomerase) positive MeSCs^9^ in upper ORS region around the bulge (UPORS) and melanocytes in the matrix were abundantly detected in Rad HF, although at a slightly reduced level (Fig. 6f). This indicated that initial melanocytes production was not disrupted by the LIR treatment. In late anagen, nearly all DCT + cells were lost from both the matrix and UPORS region of Rad HF but not noRad HF (Fig. 6g), suggesting that both MeSC maintenance and matrix melanocyte maintenance were defective in Rad HF. Interestingly, both defects were ameliorated by the PFTα treatment (Fig. 6f, g), suggesting that P53 inhibition could help to preserve not only matrix melanocytes but also MeSCs. This may be reminiscent to the elevated hallmarks of P53 pathway activation observed in the HFSCs of graying hsHFs (Fig. 5e, f).

Together, these data suggest that pharmaceutical inhibition of the P53 pathway is an effective approach to suppress hair graying. Intriguingly, the PFTα treatment also attenuated several other LIR aging phenotypes, including impaired MK sheet migration capability in skin exoplant assays and depletion of HFSC population (Supplementary Fig. S6b, c), implicating that PFTα may also help to alleviate overall skin aging to some extent.

## Discussion

HF biology was extensively delineated in mouse but not well characterized in human. Here we conducted scRNAseq analysis of highly purified hsHFs without contamination from IFE/SG/dermal cells. These high-quality data allowed us to delineate the lineage trajectory of hsHF in unprecedented detail. Our data show that hsHF contains at least 10 distinct and robustly reproducible epithelial cell groups, which are much more than that described in a previous scRNAseq study of whole human scalp micrografts that is a mixture of many tissue types^20^. Part of this striking difference likely attribute to the molecular similarity between hsHF’s middle-upper part cells and human IFE cells. There is no definitive marker to distinguish them, and they are very difficult to separate from each other by merely clustering (Supplementary Fig. S2c). With our high-quality data, we uncovered a number of novel features for hsHF, including Vimentin and FOS expression in hHFSCs, KRT10 expression in upper ORS, an innerbulge-like cell group in lower ORS and lack of a discrete companion layer cell group. These findings provided important new insights into the organization and regulation of hsHF.

Our data show that the epithelial lineage of hsHF contains two major branches: One is a matrix branch (Mx) derives from highly proliferative TACs and contains distinct cell groups resembling IRS plus multiple hair shaft lineages including cortex, medulla, and fibre cuticle; The other one is a non-matrix (noMx) branch contains quiescent HFSCs that are closely linked ORS cells. These basic patterns resemble that of mouse HF^10–13^. However, the noMx branch has several unexpected features that are not found in mouse. One unexpected feature is the IBL group that represents the lower ORS region of hsHF. It not only expresses the typical ORS marker *KRT14* but is also enriched for companion layer marker *KRT75* plus *KRT6C* and mouse bulge SC marker *SOX9* plus *LHX2*. Its closest counterpart in mouse HF would be the “Inner Bulge” cells that are ORS derivatives enriched for K6 and SOX9 expression^33^. In mouse, the “Inner Bulge” is only present in resting but not growing HFs. It provides physical anchorage for hair shaft and also a niche environment for maintaining mHFSC quiescence^33^. We speculate that the IBL compartment of hsHF also helps to provide a physical anchorage for the hair shaft and also helps to limit the proliferation of migrating hHFSC progenies that need to travel through the IBL region for a long distance before entering matrix. Consistent with this idea, *FGF18*, a key Inner Bulge derived signal for maintaining mHFSC quiescence^33^, is also enriched in the IBL compartment of hsHF (Supplementary Fig. S1e). We speculate that this structure helped hsHFs to sustain much longer anagen duration and hair shaft length than mouse HFs. Another unexpected feature in the noMx branch is the robust expression of early IFE differentiation marker KRT10 but in upper ORS regions. Although interesting, the functional importance of early IFE differentiation genes in hsHF still needs further investigation.

Surprisingly, we could not identify a clear companion layer (CP) cell group in either Mx or noMx lineage. Transcript of the classic CP marker *KRT75* (*K6hf*)^64^ is enriched in IRS and a subset of IBL cells (Fig. 1d). Consistently, while IF staining of KRT75 detected a continues CP layer from SG to matrix in anagen mouse HFs (Supplementary Fig. S1j), its signal was restricted to lower ORS and IRS areas of anagen hsHFs (Fig. 1i). In addition, another previously reported human CP marker *CALB2*^65^ was also found to be expressed mostly in IBL cells (Supplementary Fig. S1e). Based on these, we speculate that hsHF does not have a discrete CP cell group. Instead, it was the IRS cells and their nearby IBL cells that formed a KRT75 high CP like layer in their contacting interface.

Similar to mHFSCs, hHFSCs are relatively quiescent cells embedded in the upper ORS region below SG and are enriched for KRT15 and COL17A1 expression. However, most hHFSCs lack detectable expression of SOX9, a master TF for mHFSC fate^32,41,42^. This is consistent with previous reports that SOX9 marks a distinct cell population from KRT15^+^ HFSCs in hsHF^66^, suggesting significant differences in the transcriptional regulation of hHFSCs and mHFSCs. Interestingly, our data show that hHFSCs are enriched for EMT features including VIM expression, which is not present in mHFSCs. Coincidently, our transcriptional regulation network analysis identified a central regulatory role in hHFSCs for AP-1 family TFs especially *FOS*, which can also drive EMT in epithelial cells^67,68^. Notably, while normally recognized as an oncogenic process in adult epithelial tissues, EMT has been found to promote the stemness of normal mammary stem cells^69–71^. Whether EMT also functions to promote the stemness of hHFSCs would be an interesting topic for further investigation.

Hair graying was attributed to melanocyte depletion, but the underlying mechanisms were not fully understood. In human, graying hair differs from their pigmented peers in not only their color, but also growth and biophysical properties^5–7^, indicating changes in HF epithelial lineage. The HF melanocyte lineage undergoes a regeneration-degeneration cycle that is synchronized with the HF epithelial lineage^72^, which provides essential niche environments to guide the fate of the melanocytes^8,9^. MeSCs reside with HFSCs while mature melanocytes reside with matrix TACs. At anagen, HFSC activation induces MeSCs activation. At catagen, mature melanocytes undergo apoptosis along with matrix TACs. Although it is still unclear how exactly the melanocyte apoptosis is induced, it would be conceivable if it is related to the loss of matrix TACs that forms their niche environment. Here we found that graying hsHFs have significant matrix TACs depletion, which resembles an early-catagen-like matrix environment that may not be optimal for melanocytes survival.

Our data show that graying hsHFs have P53 activation in matrix TACs along with their TAC depletion. This parallel with the fact that P53 is activated in catagen matrix TACs and acts as a key driver for the TAC depletion during normal mouse hair cycle^73^. How P53 is activated in the TACs is still unclear. Hair cycle transition into catagen is related to growth factor withdrawal, and P53 is shown to mediate growth factor withdrawal-induced apoptosis^74^. On the other hand, DNA damage plays a key role in HF aging^61^. It is also a classic signal to activate P53, which is shown to mediate matrix TAC depletion upon genotoxic damage^75,76^. It is possible that the P53 activation in graying hsHFs is caused by a combination of genotoxic stress and growth factor deficiency, as well as other uncharacterized factors. Notably, graying hsHFs displayed elevated hallmarks of P53 pathway activation in not only TACs but also HFSCs (Fig. 5e, f). Meanwhile, P53 inhibition helped to preserve both matrix melanocytes and bulge MeSCs in LIR mice (Fig. 6f, g). It is likely that P53 activation in the bulge also contributed to hair graying by disrupting the maintenance of MeSCs. The role of P53 in hair graying may be a synergistic effect of its impacts on both short-term melanocyte maintenance and long-term MeSC maintenance.

At both lineage composition level and gene expression level, the differences between graying hsHF and their black neighbors were consistently observed in both relatively young and old individuals, but were not observed between hsHFs from old and young individuals, suggesting that hair graying and HF aging are not governed by identical mechanisms. This is reminiscent to the common knowledge that hair graying in human is not always linked with aging. That said, hsHF samples from different individuals displayed significantly variations in their lineage compositions likely due to individual differences, suggesting that a much bigger sample size would be necessary to accurately delineate aging-related hsHF changes among different individuals.

Notably, a previous study reported that constitutive P53 knockout in mouse did not prevent full-body IR-induced hair graying^77^. However, potential change in the magnitude of the hair graying phenotype was not thoroughly examined. In addition, constitutive P53 knockout can affect long-term HF homeostasis^73^ and full-body IR can severely impair overall health of the mice. These may have also complicated the results. Here, we carefully examined the functional importance of P53 pathway in IR-induced mouse hair graying using our LIR protocol, which induces premature HF aging and hair graying in a restricted skin area without significantly impairing overall mouse health. Our data showed that the LIR hair graying was associated matrix TAC depletion and matrix P53 pathway activation. These were consistent with our findings in graying hsHFs. Importantly, therapeutic inhibition of P53 pathway in the LIR mice significantly reduced, although did not abolish, their matrix TAC depletion and hair graying phenotypes, supporting the functional importance of P53 pathway in the development and treatment of hair graying. That said, we are aware that the hair graying induced by LIR aging is not completely identical to human hair graying. For example, the LIR aging caused HFSC depletion which was not observed in our human hair graying data. Clinical studies would be important to determine if P53 blockade could alleviate human hair graying and potentially other aspects of HF aging in vivo.

## Methods

### Ethic of human samples

hsHF samples were from discarded plastic surgery specimens from healthy individuals with written informed consent. The procedures were in accordance with protocols approved by the Board of Ethical Review of Shanghai Ninth People’s Hospital and Ethics Committee of Shanghai Institute of Nutrition and Health, and were in accordance with the Helsinki Declaration of 1975, as revised in 1983.

### Human HF separation

Human scalp samples were digested in 4 mg/ml DispaseII (Sigma Aldrich) overnight. Individual HFs were then pulled out one by one using forceps. To obtain single-cell suspension for scRNAseq, the isolated HFs were digested in 0.05% Trypsin-EDTA (Gibco) for 30 mins at 37°C, washed in PBS + 1%FBS + 1 mM EDTA, and filtered through a 100 μm cell strainer.

### Animals

All animal procedures were performed in accordance with protocols approved by Institutional Animal Care and Use Committee (IACUC) of the Shanghai Institute of Nutrition and Health, Chinese Academy of Sciences. WT C57BL/6 J mice were purchased from Shanghai SLAC Laboratory Animal Co. Ltd. Female mice were used in all studies. For hair color quantification, mouse hairs within a 5 mm x 5 mm area of the target region were plucked by forceps, placed on glass slide and counted under stereomicroscope (Nikon, SMZ745T).

### Localized irradiation

Localized irradiation was achieved using tinplates each carrying a 2 cm diameter hole to restrict IR area to mouse upper back while protecting the other body regions. X-ray irradiation was conducted by Xstrahl-CIX3 (300 kV/10 mA) for a total dosage of 10 Gy at Shanghai Institute of Nutrition and Health, Chinese Academy of Sciences. 0.001 g/ml Neomycin (Sangon, A610366-0100) was administrated in the drinking water of the LIR mice after irradiation for 1 month.

### Topical administration of drugs

PFTα (Selleck, S2929) was dissolved in dimethylsulfoxide (DMSO; Thermo Fisher Scientific) at 20 mg/ml stock concentration respectively. For topical application, the stocks were mixed 1:50 with a solvent solution containing 50% ethanol, 30% water, and 20% propylene glycol. The mixture was topically applied to mice at 2 μg/cm_2_ dosage every 3 days for 5 times post IR.

### Immunofluorescence (IF) staining

For IF staining of sections, cryosections were made from frozen tissues embedded in the OCT compound (Tissue Tek). Slides were fixed for 10 mins in 4% paraformaldehyde and blocked for 1 hr in blocking buffer (2.5% normal donkey serum + 2.5% normal goat serum + 1% BSA + 0.3% Triton X-100). Sections were then incubated with primary antibodies at 4 °C overnight and with fluorochrome-conjugated secondary antibodies at room temperature for 1 h. Slides were then washed in PBS and mounted with Fluoromount-G mounting media (Invitrogen). Images were taken using Zeiss Cell Observer System (Zeiss). For whole-mount IF staining, the human scalp was digested in 4 mg/ml DispaseII (Sigma Aldrich) overnight, plucked by forceps on the second day, and stained using the same procedures described above. Z-stack images were taken using Zeiss Cell Observer System (Zeiss). The following antibodies dilutions were used: KRT14 (rabbit, Zhang lab, 1:1000), KRT14 (chicken, Biolegend, 1:200), KRT10 (rabbit, Covance, 1:1000), K6hf (rabbit, Zhang lab, 1:1000), Loricrin (rabbit, Covance, 1:1000), SOX9 (rabbit, Abcam, 1:200), CD49f (rat, Biolegend, 1:200), Ki67 (rabbit, Abcam, 1:1000), COL17A1 (rabbit, Abcam, 1:200), c-KIT (rat, Biolegend, 1:200), γH2Ax (rabbit, CST, 1:500), KRT15 (mouse, Santa Cruze, 1:200), CD200 (rat, Biolegend, 1:200), LHX2 (rabbit, Abcam, 1:200), FOS (rabbit, CST, 1:200), LGR5 (rabbit, Abcam, 1:200), TCHH (mouse, Santa Cruz, 1:500), LEF1 (rabbit, CST, 1:500), P21 (rabbit, CST, 1:500), VIM (rabbit, Abcam, 1:200), TRP1(mouse, Santa Cruz,1:500), GATA3 (rat, eBioscience, 1:500), DCT(rat, gift from Ting Chen lab, 1:200).

### Exoplant culture

Mouse backskins were harvested after hair removal using Veet depilatory cream. Subcutaneous fat was removed with a scalpel. 1.5 mm biopsy punches from the samples were placed with 1 μL Matrigel (Corning) on fibronectin (Millipore) coated 24 well culture dishes. CNT-Prime medium (Cellntech) was used for culturing. Images were taken using Zeiss A1 microscope (Zeiss).

### FACS analysis

After removing subcutaneous fat with a scalpel, the skins were digested in Trypsin-EDTA at 4 °C overnight. Epithelium cells were then released by scraping the epidermal side and filtered through 40 μm cell strainers. CD34 (eBiosciences) and CD49f (Biolegend) were incubated for 30 min on ice. FACS analyses were performed using BD CytoFLEXLX (BD Biosciences) and analyzed with FlowJo 7.6 software.

### Statistical analysis

Comparisons between two groups were performed using an unpaired two-tailed Student’s *t*-test. Quantitative data displayed as histograms are expressed as means ± standard error of the mean (represented as error bars). Graphpad Prism software and Excel (Microsoft) were used to assess statistical significance. Statistical significance was set at a *P* < 0.05. Cell number comparisons in Fig. 5a, were normalized to the total cell numbers of each sample. For example, the F62 IBL bar represents (IBL % in F62B)/(IBL % in F62W). The same applies to all similar figures in this manuscript.

### scRNA-seq data analysis and cell-type identification

We used the Seurat R package (version 3.2.2)^22^ to further analyze the scRNA-seq data. After the initial Cell Ranger metric assessment, cells with fewer than 800 genes or more than 5000 genes detected, and more than 10% mitochondrial genes were further excluded from the downstream analyses. After quality control, 14,169 cells remained and were used for downstream bioinformatic analyses (See Supplementary Fig. S7a for QC metrics). We used “sctransform” framework for the normalization and variance stabilization of molecular count data from scRNA-seq experiment. This procedure omits the need for heuristic steps including pseudocount addition or log-transformation and improves common downstream analytical tasks such as variable gene selection, dimensional reduction, and differential expression.

To avoid batch effects among samples and experiments, the F18, F59, F62B, F62W samples were first integrated into a reference dataset by CCA method using the “FindIntegrationAnchors” function of Seurat. The top 1800 highly variable genes in these samples were used to identify integration anchors. Next, Dimensionality reduction was performed with “RunPCA” function and the PCs were then used for downstream dimensionality reduction and clustering analyses. Total cell clustering was performed by “FindClusters” function at a resolution of 0.3 and the first 30 PCs (determined by “Elbow plot”) were used to define cell identity. Dimensionality reduction was performed with “RunUMAP” function and visualized by Uniform Manifold Approximation and Projection (UMAP)^78^. F31B and F31W samples were treated as query datasets. Cells in the query datasets were projected into the clusters of the reference dataset and visualized on the reference UMAP structure via the “MapQuery” function of Seurat package (Supplementary Figs. S7b, c).

For subgroup cell fig clustering, cells of different types were extracted separately and clustered by their respective first 15 PCs (determined by “Elbow plot”) using resolutions of 0.15 for HFSCs and 0.2 for PRs. To further approximate the low-dimensional data manifold representing the differentiation trajectory for PR cells, the first 15 PCs were used to create the diffusion maps through R package destiny (version 3.0.1)^53^. Marker genes for each cluster were determined with the Wilcoxon rank-sum test by “findMarkers” function implemented in the scran R package (1.14.5)^23^. Only those with average ‘AUC’ (Area under curve) > 0.7 and ‘FDR’ < 0.01 were considered as marker genes. Marker genes for each cluster are shown in Supplementary Table S2. Annotation of immune cell identities was conducted using the SingleR^57^. For gene set score analysis, we used the “AddModuleScore” function to calculate module scores for gene sets (Marker genes) in single cells. Ribosome Gene set were obtained from the MSigDB database (KEGG). Genes in each gene set are listed in Supplementary Table S4.

### Integration of previous human eyelid skin scRNAseq data with our hsHF data

We download the published dataset^51^ of human skin samples from Aging Atlas (https://bigd.big.ac.cn/aging/, accession number: HRA000395). The raw data first went though the same data preprocessing pipeline as the hsHF data. Then cell group labels were assigned based on the markers described in the publication^51^. The data were then integrated with hsHF data and clustered into UMAP using Seurat with top 1200 highly variable genes and reciprocal PCA (‘RPCA’) method^22^.

### Identification of white hair follicle Associated DEGs

We used the function of “FindMarkers” in Seurat to identify white-vs-black differentially expressed genes (DEGs) for each cell type. The log fold change and adjusted *p* value of each DEG were calculated by using the non-parametric two-sided Wilcoxon rank-sum test and only those with ‘avg_logFC’ > 0.05’, ‘min.pct’ = 0.2 and ‘p_val_adj’ < 0.05 were considered to be associated DEGs. The results were listed in Supplementary Table S6.

### Identification of age-associated DEGs

To identify age-dependent DEGs, we used the function of “FindMarkers” in Seurat to identify age-dependent DEGs of different ages (F62B-vs-F31B; F59-vs-F18). The log fold change and adjusted *P* value of each DEG were calculated by using the non-parametric two-sided Wilcoxon rank-sum test and only those with ‘avg_logFC’ > 0.05’, ‘min.pct’ = 0.2 and ‘p_val_adj’ < 0.05 were considered to be associated DEGs.

### RNAseq of mouse skin epithelium and data analysis

As previously described^62^, mouse backskin samples were washed in PBS, cut to 5 mm^2^ pieces, submerged into 4 mg/ml DispaseII (Roche, diluted in PBS) in 1.5 mL Eppendorf tube and placed in 37 °C water bath for 1 h. After digestion, the skin epithelium was isolated by forceps, washed twice in PBS.

Total RNA samples were extracted from the isolated skin epithelium (*n* = 3 each) using the miRNeasy Mini kit (QIAGEN). Illumina RNA-seq were conducted by the Novagene company following standard protocol. RNA-seq data were processed by the Salmon 1.2.0 software using default setting as previously described^62^. Differentially expressed genes (DEGs) were then calculated using the DESeq2 package (version 1.2.4)^79^. The DEGs are listed in Supplementary Table S9. For cross-species GSEA analysis, mouse and human homolog genes were mapped together based on identical gene symbols. Only the mouse genes that have detectable human homolog in the hsHF scRNAseq dataset were included in the cross-species GSEA analysis.

### Function enrichment analysis

GO and GSEA analysis of DEGs was performed by clusterProfiler^80^ R package (version 3.14.3) and visualized with the ggplot2 R package (version 3.3.2). Representative terms selected from the top 10 ranked GO terms, KEGG, HALLMARK terms or pathways (*P* < 0.05) from MSigDB were displayed.

### Pseudotime analysis

R package Monocle3^35^ was used to reconstruct the hair follicle cell developmental trajectory. The UMI matrix was used as input and variable genes obtained from epithelial cell types were detected by Seurat to sort cells in pseudotime. The junction of SC1 and SC2 was defined as the root state argument and aligned via the “ordercells” function. “UMAP” was applied to reduce dimensions and the visualization functions “plot_cell” were used to plot each group along the same pseudotime trajectory.

### Transcriptional regulatory network analysis

The transcriptional regulatory network was analyzed by the GENIE3^81^ (version 1.8.0) and RcisTarget^54^ (version 1.6.9) R packages of the SCENIC (version 1.1.2.2)^54^ workflow using default parameters. Transcription factors (TFs) of hg19 were used as reference TFs and downloaded using RcisTarget. The gene expression matrix passed the default filtering for each sample were normalized from Seurat as input. First, co-expression modules between transcription factors (TFs) and the potential target genes were identified based on the gene expression matrix through GENIE3. Second, for each co-expression module, the cis-regulatory motif enrichment analysis was performed among all potential target genes by RcisTarget, and only the target genes enriched with the motifs of the corresponding TFs were selected as direct target genes. Each transcription factor and its direct target genes were defined as a regulon. Then, we scored the regulons in the cells with AUCell (version 1.8.0)^54^. Finally, we remove the regulons if the correlation between regulons and TFs was lower than 0.25 or the TFs’ expression was low (<0.6) in all cell types. Networks of the TF modules were visualized by Cytoscape^82^ (version 3.7.1).

### Characterization of cell fate probabilities

We used Palantir^52^ to further characterization of cell fate probabilities for MSX2^+^ TA cells. Palantir is a tool that, using pseudotime distances, identifies trajectory endpoints (‘terminal cells’) in data of differentiating cells and, moreover, measures entropy in cell phenotypes to measure their plasticity (‘differentiation potential’) and commitment to specific cell fates (‘branch probability’). Four terminal state cells were defined by choosing the cells from CO, IRS, FC, and ME, respectively. An initial cell was defined by choosing the cells with the lowest signature score of MxMkr in *MSX2*^+^ MxTA cells. The number of neighbors was set to *k* = 20, and the number of diffusion components was set to 5. For all other parameters, default settings were used.

## Supplementary information

Supplementary Information

Supplementary table S1

Supplementary table S2

Supplementary table S3

Supplementary table S4

Supplementary table S5

Supplementary table S6

Supplementary table S7

Supplementary table S8

Supplementary table S9

## Data Availability

scRNAseq data have been deposited online at the following site: https://www.biosino.org/node/project/detail/OEP002321.

## Code availability

The code for processing scRNAseq datasets in the paper is available at: https://github.com/zhendejuzi/scRNA_HF. Alternative site: https://gitee.com/wusijie1990/scRNA_HF.
