# Supplementary material for: Single-cell transcriptomics reveals lineage trajectory of human scalp hair follicle and informs mechanisms of hair graying
Source: Cell Discov. 2022 May 24;8:49. doi: 10.1038/s41421-022-00394-2 (PMC9126928; doi:10.1038/s41421-022-00394-2)
Supplement: Supplementary file 1 — Supplementary Information [file 41421_2022_394_MOESM1_ESM.pdf]

Supplementary Information

Supplementary Fig. S1

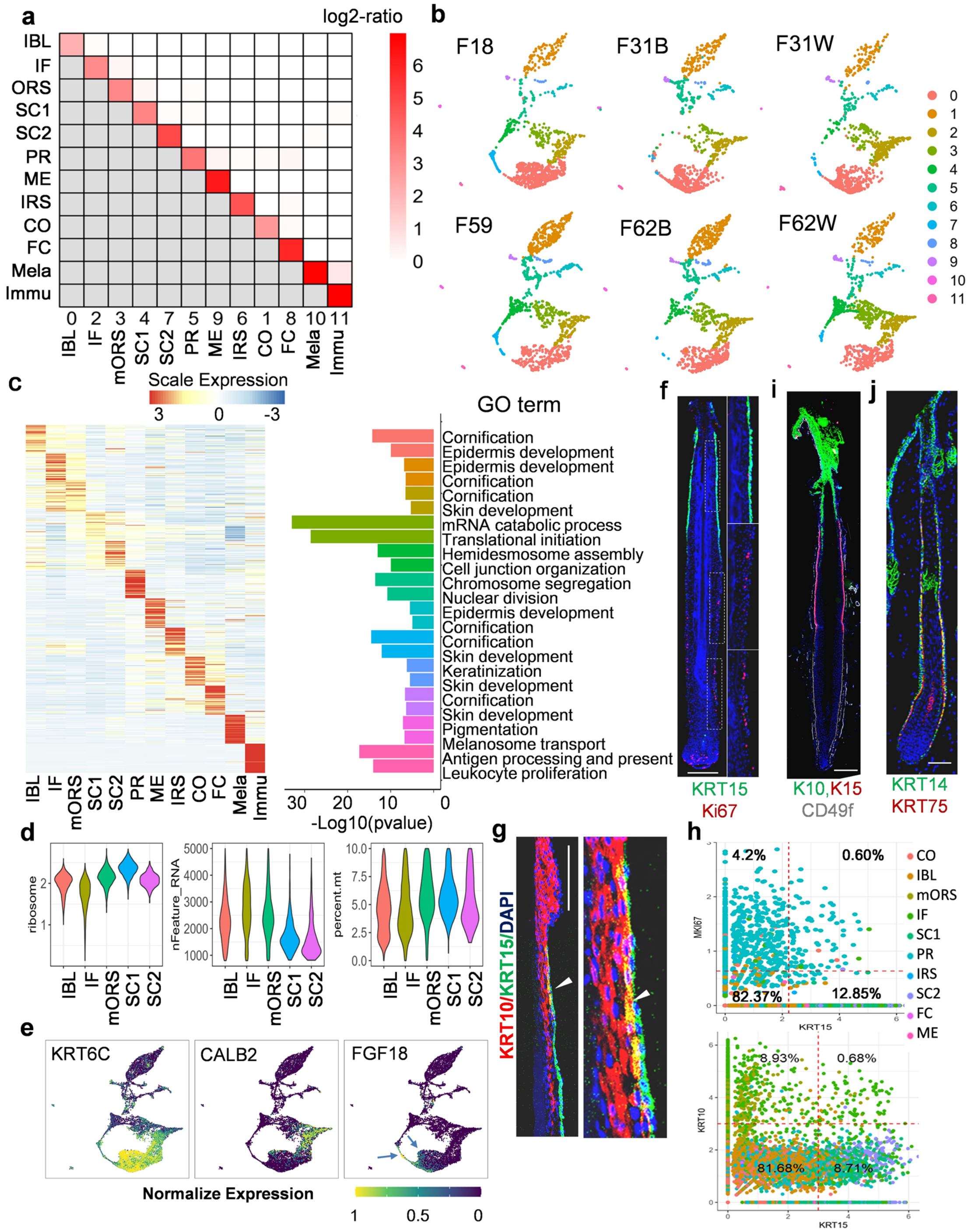

Supplementary Fig. S1: Single-cell RNA-seq reveals cell type composition of human scalp hair follicle.

a) Heatmap of the log2-ratio of the total weight between nodes in the same cluster or in different clusters, relative to the total weight expected under a null model of random links. Grids in the lower left half were manually set gray since they were duplicates of upper right grids. **b**) UMAP plot showing 12 cell types of human scalp hair follicle in each of the 6 samples. **c**) Heatmap showing gene expression signatures of each cell type. Left, each column represents one cell type and each row indicates the expression of one gene; the size of the dot represents cell number and the value for each gene is row-scaled Z score. Enriched GO terms for each cell type are to the right. **d)** Violin plot showing the expression of ribosome genes (KEGG term), 'nFeature_RNA' and 'percent.mt' across different cell types. **e)** Representative marker genes expression visualized on UMAP of hsHF. Arrows indicate positive cells in a graph. **f**) Representative IF images (n=5 HFs from 3 individuals, same as below) of human scalp HF sections and magnified views of the squared areas. Scale bars: 200µm. **g)** Representative IF images (n=5) of the upper ORS region of human scalp HF sections (left) and a magnified view of the middle region of the left picture (right). Scale bars: 100µm. Arrow: KRT10+KRT15+ cells. **h**) Upper panel: 2D-plot of *KRT15* (x-axis) and *MKI67* (y-axis) expression. Each dot is a single cell. Cell groups are colored as indicated on the right. Dashed lines represents high expression criteria for MKI67 (horizon, y=0.69, median expression level of *MKI67* in PR cells) and for KRT15 (vertical, x=2.30, top rank 10% expression level of *KRT15* in Mx cells). Numbers indicate cell percentage in each quarter. Lower panel: 2D-plot of *KRT15* (x-axis) and *KRT10* (y-axis) expression. **i)** Representative IF images (n=3 HFs from 3 individuals) of human scalp HF micrograft sections. Scale bars: 200µm. **j**) Representative (n=3) IF images of mouse anagen (2 weeks post depilation at P50) HF sections (Scale bars: 50µm). Scale bars: 50µm.

Supplementary Fig. S2

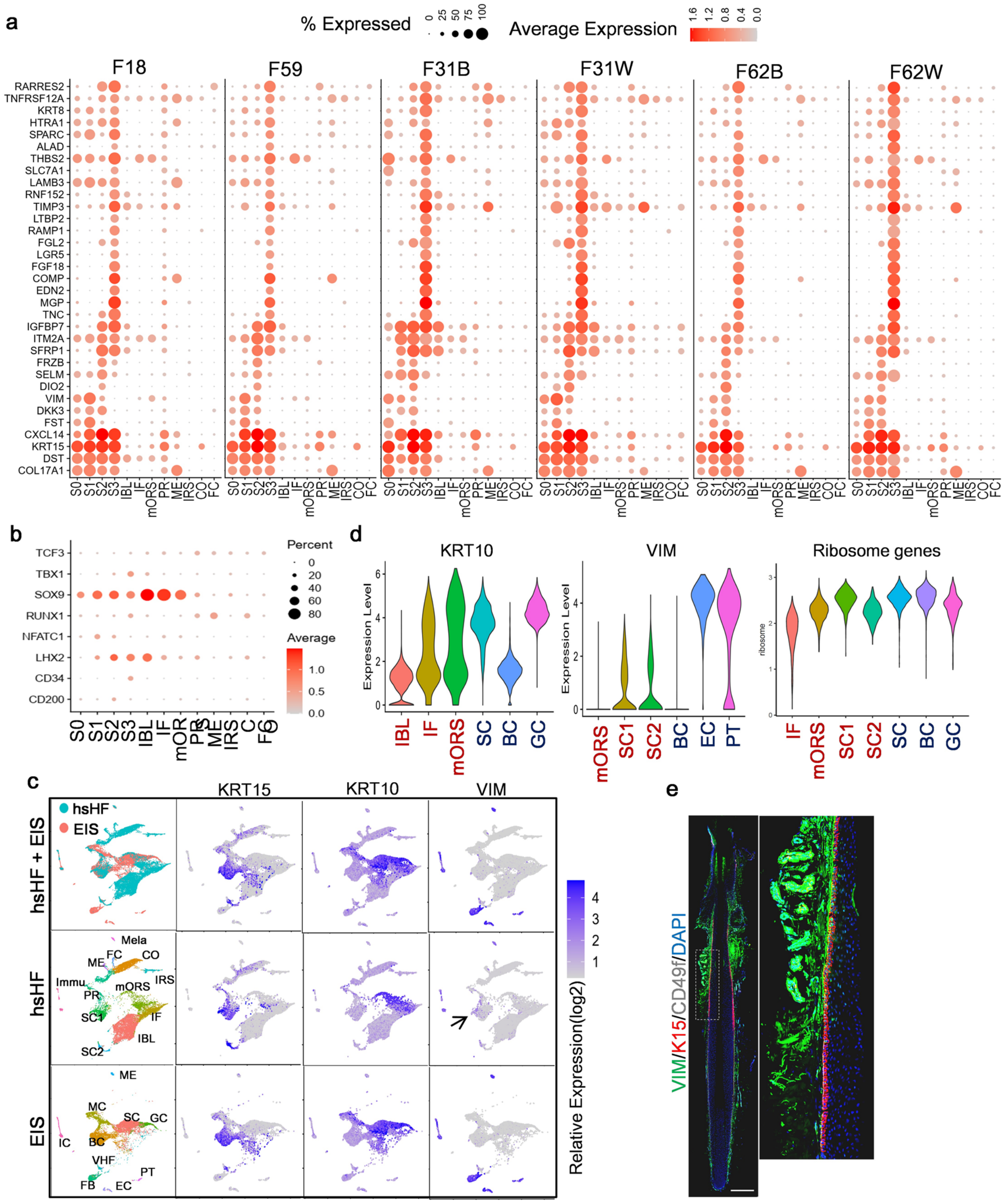

Supplementary Fig. S2: Human scalp HFSCs display different molecular features from their Murine counterparts.

a) Dot plot showing the expression of human HFSC signature genes in each of the 6 individual samples. **b)** Dot plot showing the expression of mouse HFSC signature genes across 12 cell types of human scalp hair follicle. **c)** Left column: Integrated UMAP of hsHF and eyelid skin (EIS) dataset, and separated display of hsHF and EIS cells in this UMAP . EIS cell populations were labeled according the previous publication as following: BC, Basal cell; MC, Mitotic cell; VHF, Vellus hair follicle; ME, Melanocyte; SC, Spinous cell; GC, Granular cell; EC, Endothelial cell; IC, Immune cell; Fibroblast (FB); Pericyte (PT). Right columns: marker genes expressions plotted on the UMAP as indicated. Arrows indicate positive cells in a graph. **d)** Violin plot showing the expression of *KRT10*, *VIM* and ribosome genes across different cell types as indicated. **e)** Representative (n=3 HFs from 3 individuals) IF images of human scalp HF micrograft sections and magnified views of the squared areas. Scale bars: 200µm.

Supplementary Fig. S3

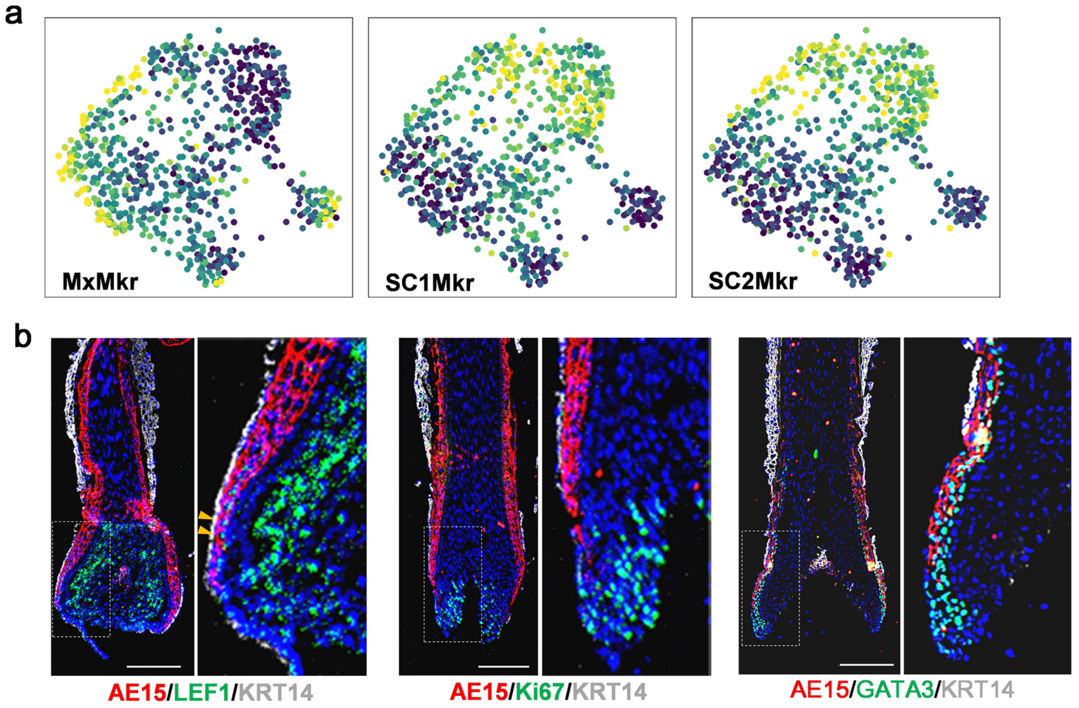

Supplementary Fig. S3: Human HF TAC contains heterogeneous populations with distinct lineage tendency.

a) Representative marker genes expression visualized on MxTA subclustering UMAP. MxMkr: Mx markers; SC1Mkr: SC1 markers; SC2Mkr: SC2 markers. **b)** Representative (n=3 HFs from 3 individuals) IF images of human black HF sections and magnified views of the boxed areas. Scale bars: 100µm. Arrow heads: cells with both LEF and AE15 expression.

Supplementary Fig. S4

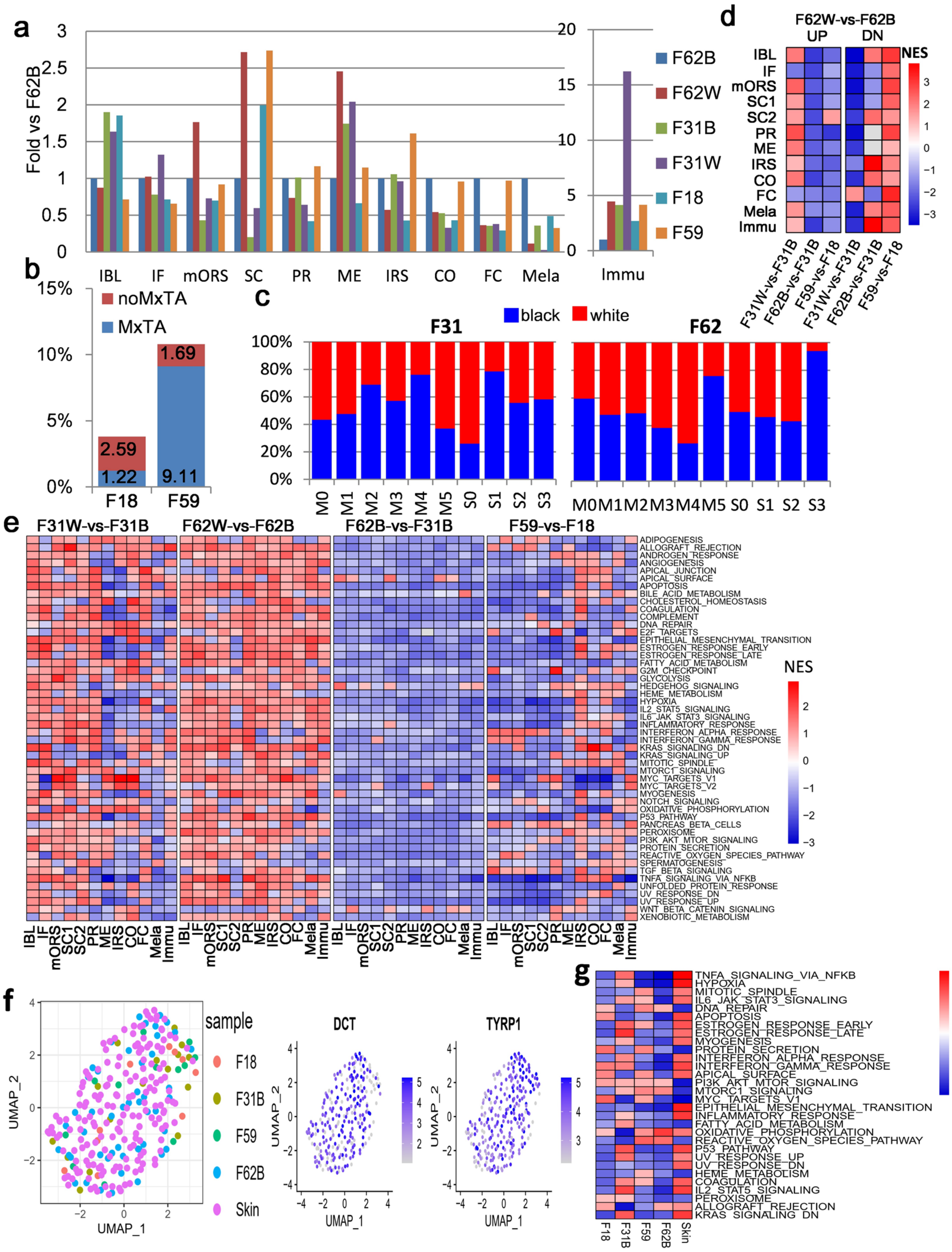

Supplementary Fig. S4: Depletion of Matrix TAC is a major HF change associates with hair graying

a) Bar plots of relative abundance of each cell population in each sample. Y axis: fold change of cell population % in each sample vs that in the F62B. **b)** Bar plots of noMxTA and MxTA cell % in all HF epithelial cells in F18 and F59. Numbers indicate exact %. **c)** Bar plots of relative abundance of each MxTA or hHFSC subgroup in white hsHFs and their black neighbors in F62 and F31 samples as indicated. **d)** Heatmap summarizing pre-ranked GSEA analysis results. Significantly up-regulated (UP) or down-regulated (DN) gene in F62W-vs-F62B comparison in each cell group (indicated on the left side) were used as the input gene sets (Input-geneset). Ranked lists of genes (Rnk) were produced using gene expression fold change in each cell group in the sample comparison pairs indicated at the bottom. Red indicated enrichment while blue indicated depletion. NES: normalized enrichment score. **e)** Heatmap summarizing pre-ranked GSEA analysis results of MSigDB hallmark terms (right side) in each cell group (bottom) of each sample comparison pairs (top). Rnks were produced as mentioned above. **f)** Left: UMAP plot of re-cluster result of melanocytes [hsHF melanocyte group (Mela) + EIS melanocyte group (ME)]. Colors indicate samples. Skin=EIS ME. Right: marker genes expression visualized on the UMAP. **g)** Heatmap summarizing pre-ranked GSEA analysis results for the relative enrichment of MsigDB Hallmarks in each of the melanocytes samples. Rnks were produced using gene expression fold change in each sample vs all the other samples.

Supplementary Fig. S5

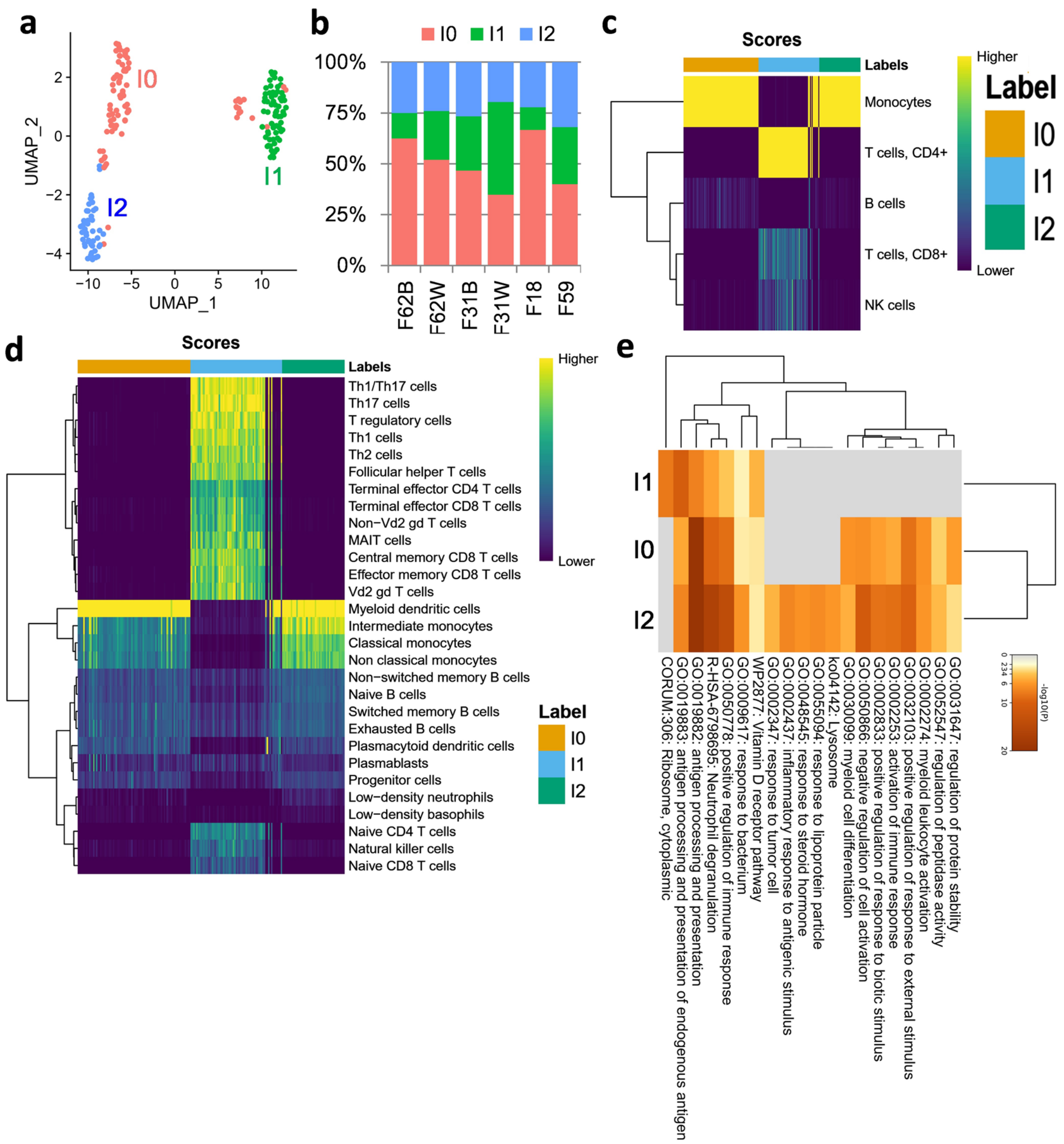

Supplementary Fig. S5: Analysis of potential immune cell changes in hair graying. **a)** UMAP plot of re-cluster result of the immune (Immu) cell group in hsHF. Different subgroups were indicated by different colors. **b)** Bar plot of immune cell subgroup composition in each hsHF sample. **c)** Heatmap of SingleR scores for the hsHF immune cells across top correlated reference labels in Database of Immune Cell Expression/eQTLs/Epigenomics (DICE)[1]. **d)** Heatmap of SingleR scores for the hsHF immune cells across top correlated reference labels in Monaco immune data[2]. **e)** Heatmap summarizing enrichment analysis results of representative GO terms across different Immu cell subtypes using Metascape[3].

Supplementary Fig. S6

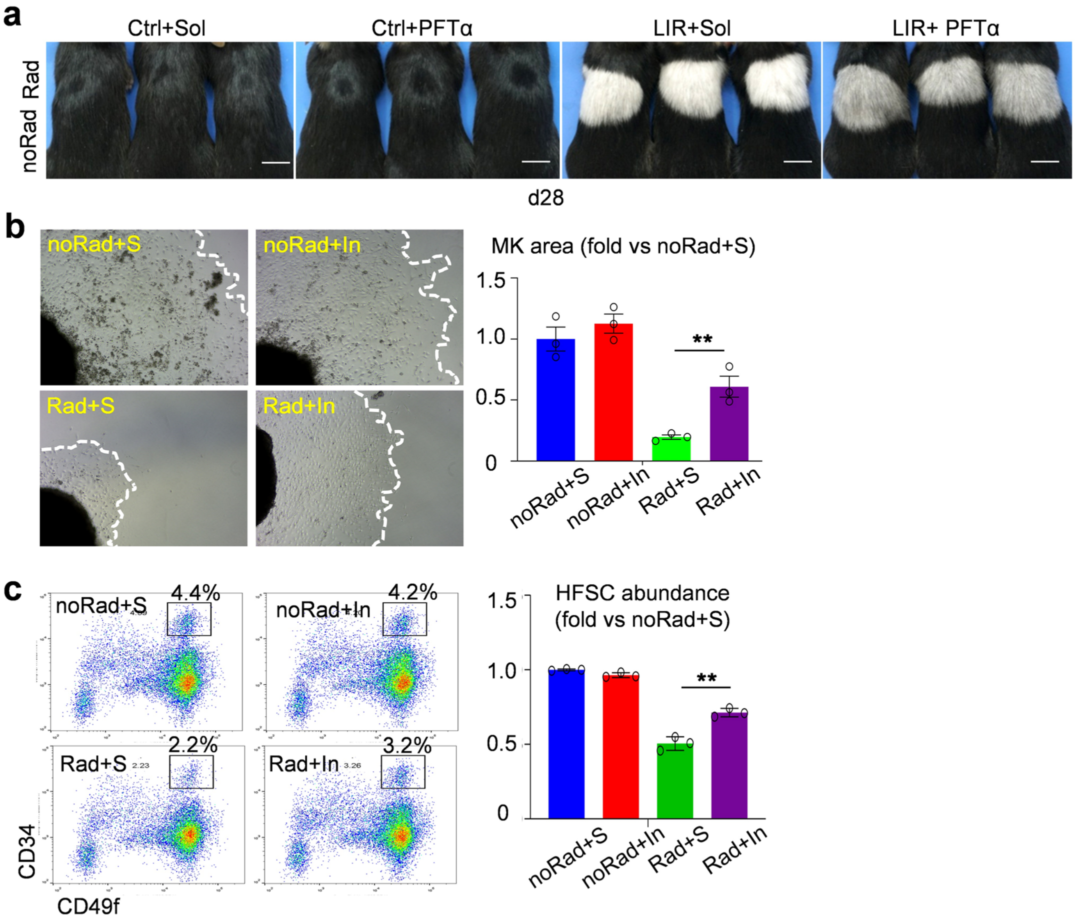

Supplementary Fig. S6: Pharmaceutical inhibition of P53 pathway ameliorate ionizing irradiation induced hair graying in mice.

a) Full backskin images of solvent control (+Sol) or PFTα (+In) treated LIR mice or control (Ctrl) mice without LIR at 4 weeks post Wax1 time. Scale bars: 1cm. **b)** Photos of day 7 exoplant cultures of the noRad or Rad skin areas of Sol (+S) or PFTα(+In) treated LIR mice backskins and quantification (n=5) of MK sheet area. Dashed lines: Edge of MK sheet. #: skin exoplant. **c)** FACS profiles of the above Sol or PFTα treated LIR mice backskins' epithelium and their HFSC abundance quantification (n=3).

Supplementary Fig. S7

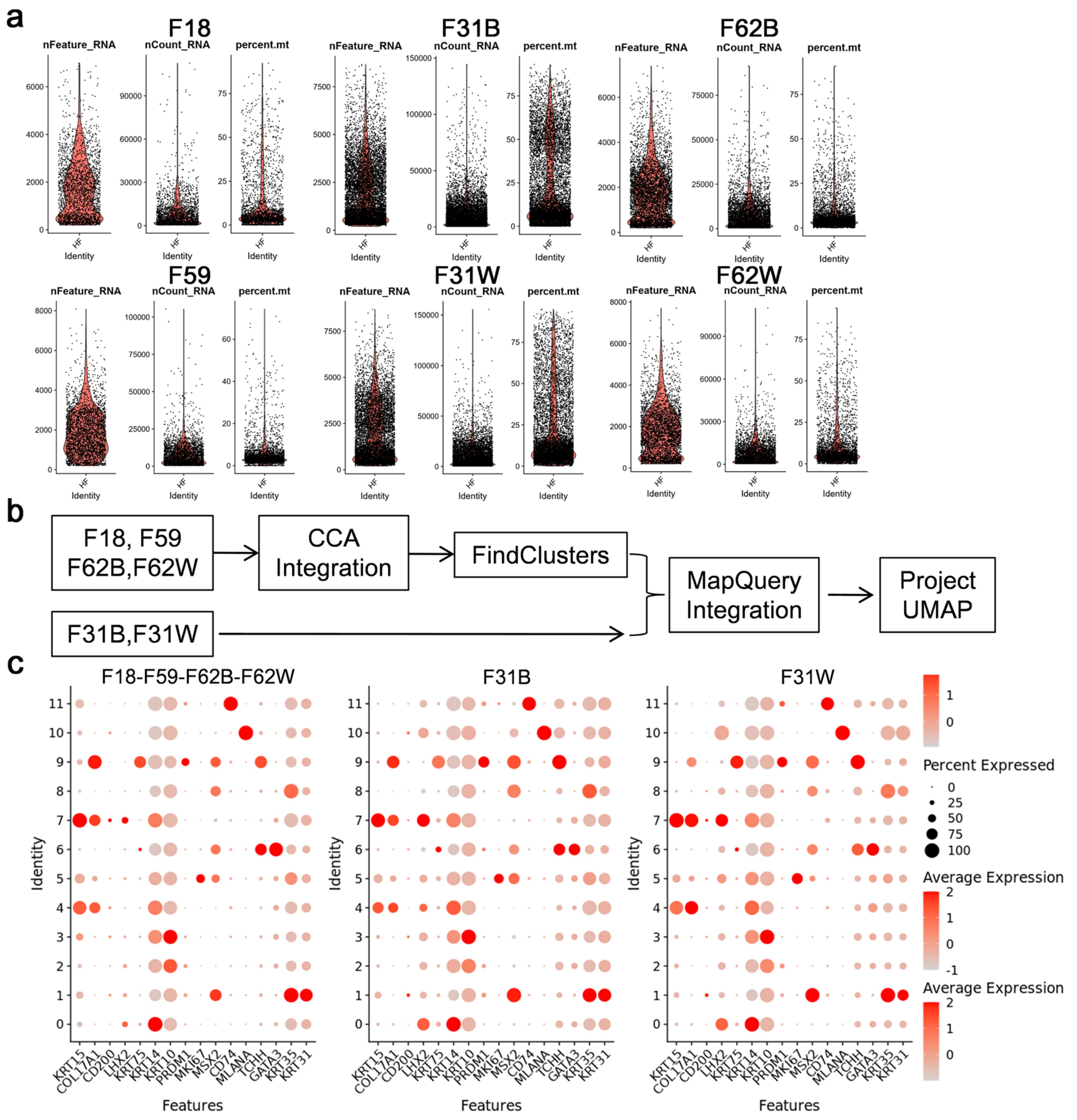

Supplementary Fig. S7: Quality control and integration of scRNA-seq data.

a) Violin plots of QC metrics of each hsHF samples. nFeature_RNA, the number of unique genes detected in each cell; nCount_RNA, the total number of molecules detected within a cell; percent.mt, The percentage of reads that map to the mitochondrial genome in each sample. **b)** Diagram of data integration process. Briefly, we first used standard Seurat package to integrate F18, F59, F62B, F62W samples via CCA method and clustered into cell groups. This is the reference dataset. F31B and F31W samples were treated as query datasets. Cells in the query datasets were projected into the clusters of the reference dataset via the “MapQuery” function of Seurat package. **c)** Dot plots showing the expression of key lineage markers in the reference dataset (F18-F59-F62B-F62W) as well as F31B and F31W samples after MapQuery projection. Vertical axis: cell clusters. It appears that the projected F31B and F31W samples have similar marker expression pattern as the reference dataset, indicating that the data integration is successful.

Reference

1 Schmiedel, B. J. *et al.* Impact of Genetic Polymorphisms on Human Immune Cell Gene Expression. *Cell* **175**, 1701-1715 e1716, doi:10.1016/j.cell.2018.10.022 (2018).

2 Monaco, G. *et al.* RNA-Seq Signatures Normalized by mRNA Abundance Allow Absolute Deconvolution of Human Immune Cell Types. *Cell Rep* **26**, 1627-1640 e1627, doi:10.1016/j.celrep.2019.01.041 (2019).

3 Zhou, Y. *et al.* Metascape provides a biologist-oriented resource for the analysis of systems-level datasets. *Nat Commun* **10**, 1523, doi:10.1038/s41467-019-09234-6 (2019).
